# Cas10 residues lining the target RNA binding channel regulate interference by distinguishing cognate target RNA from mismatched targets

**DOI:** 10.1080/15476286.2026.2633385

**Published:** 2026-02-18

**Authors:** Sarah A. Khweis, Mason A. Blackburn, Calvin C. Perdigao, Megan O. Pierce, Colby R. Lewis, Jack A. Dunkle

**Affiliations:** Department of Chemistry and Biochemistry, University of Alabama, Tuscaloosa, AL, USA

**Keywords:** CRISPR-Cas, Cas10, cyclic oligoadenylate, interference, type III

## Abstract

Type III CRISPR systems are defined by the presence of the Cas10 protein and are among the most abundant CRISPR systems in nature. Cas10 forms a complex with crRNA and several Cas proteins that surveils prokaryotic cells for foreign RNA molecules and when they are detected it activates a cascade of interference activities. The synthesis of the cyclic oligoadenylate signalling molecule by Cas10 is a key aspect of the interference cascade. Despite structures of the Cas10 complex bound to target RNAs, the molecular mechanism by which Cas10 senses the bound state to licence interference is lacking. We identified five residues in *S. epidermidis* Cas10, two in the Cas10 Palm2 domain and three in domain 4, that line the target RNA binding channel. We assessed the contribution of these residues to interference in the context of a cognate or mismatched target RNA. We found that the residues regulate whether a mismatched crRNA-target RNA duplex is able to activate interference *in* *vivo*. We purified two site-directed mutants of Cas10-Csm and show with *in vitro* cOA synthesis assays that they demonstrate enhanced discrimination of cognate versus mismatched target RNAs.

## Introduction

CRISPR-Cas systems, present in approximately 85% of archaea and 40% of eubacteria, protect these organisms from foreign genetic elements and predation by bacteriophage by detecting the presence of a foreign nucleic acid and mounting an interference response [1,2]. Several steps in this process are common to all CRISPR-Cas systems including crRNA biogenesis, assembly of a ribonucleoprotein complex containing a crRNA bound to a Cas effector protein and surveillance of the cell for nucleic acids that can base-pair to the crRNA which activates interference [3]. However, important differences also exist. Some systems sense foreign DNA while others sense RNA and the architecture of the crRNA-bound effector complex varies substantially.

CRISPR-Cas systems have been grouped into two classes and seven types [4]. Classes 1 and 2 are distinguished by whether the crRNA-bound effector is a multi-protein complex or a single protein. The *S. epidermidis* Cas10-Csm complex is class 1 and type III-A. These complexes recognize foreign RNA that can base-pair with the crRNA. The base-pairing event stimulates synthesis of second messenger molecules, cyclic oligoadenylates (cOA), and these molecules bind to ancillary proteins which contribute to interference via RNase, DNase or other activities [5–10].

**Figure 1.**
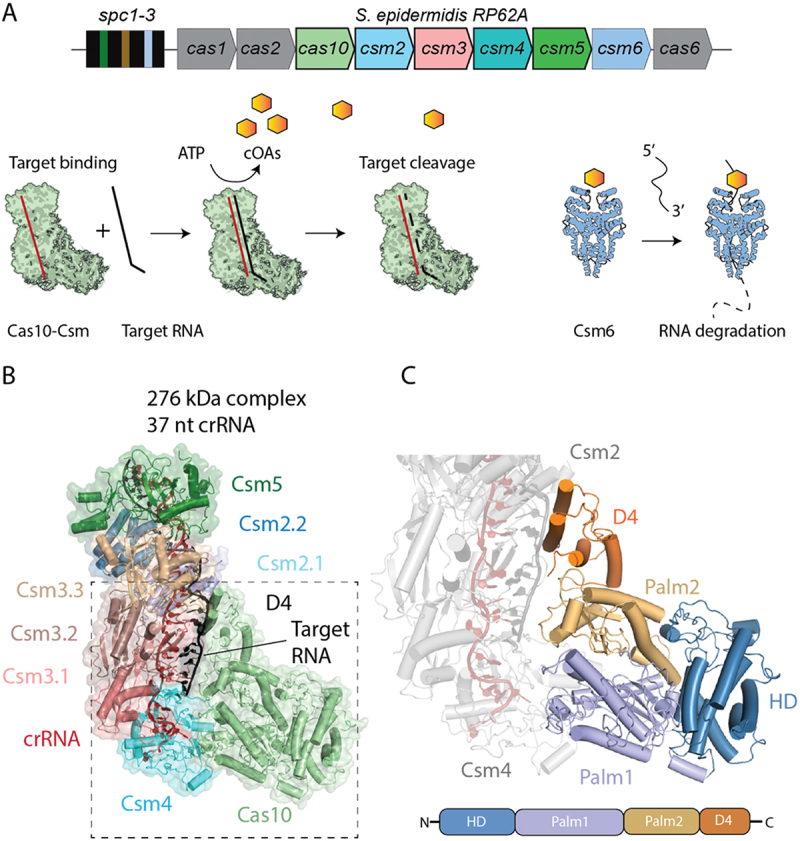
The structure and activity of the Cas10-Csm complex. (A) The *CRISPR-Cas10* locus of *Staphylococcus epidermidis* strain RP62A contains three spacers which after transcription and maturation by the Cas6 RNase become crRNAs. Each crRNA associates with Cas10 and Csm2-5 proteins to form the Cas10-Csm ribonucleoprotein complex. This complex can detect foreign RNAs which activates cyclic oligoadenylate (cOA) synthesis. COAs bind to Csm6 and elicit interference by degrading RNA. (B) the Cas10-Csm complex contains a single copy of Cas10, Csm4 and Csm5. *In vivo* a 276 kDa complex is formed containing three Csm3 proteins, two Csm2 proteins and a 37 nt crRNA [11]. A 318 kDa complex also forms containing a 43 nt crRNA and an extra copy of Csm2 and Csm3. (C) Cas10 is composed of an HD domain which possesses single-stranded DNase activity in many Cas10, a Palm-polymerase like domain – Palm1—fused to a Zn-finger containing region, a Palm2 domain that possesses the active site for cOA synthesis and the C-terminal domain 4 (D4).

The *S. epidermidis* Cas10-Csm complex (SeCas10-Csm), a type III-A CRISPR system, typifies these features. SeCas10-Csm is an approximately 276–318 kDa complex formed of Cas10, Csm2 (Cas11), Csm3 (Cas7), Csm4 (Cas5) and Csm5 bound to a crRNA of length 37 or 43 nucleotides (Figure 1(A)) [11,12]. The crRNA interacts extensively with the Csm3, Csm4 and Csm5 proteins (Figure 1(B)). Target RNA, that is RNA with complementarity to the crRNA, can form a duplex with it. The Cas10 and Csm2 proteins extensively interact with the bound target RNA and substantial conformational changes occur in the complex upon target binding (Figure 1(B,C)) [11,13–15]. CA_6_, a cOA made of six adenosine monophosphate subunits, is synthesized by Cas10 and this second messenger binds to Csm6 activating its latent RNase activity [16,17], an activity that provides potent interference against foreign plasmids and bacteriophage (Figure 1(A)) [16–19].

The biological role of Cas10-Csm is to detect the presence of foreign RNAs with sensitivity and specificity and couple this detection event to molecular cascades to block replication of foreign nucleic acids. Therefore, molecular diagnostic assays can be built upon the Cas10-Csm complex by simply programming it with a crRNA with complementarity to a pathogen RNA and coupling the molecular cascade to a desirable chemistry for read-out [20–23]. For example, Nemudraia and colleagues showed that the intrinsic signal amplification of Cas10-Csm could be leveraged to detect SARS-CoV-2 RNA at femtomolar sensitivity, equivalent to lateral flow strip diagnostics for SARS-CoV-2, without the use of specialized antibodies or PCR amplification [24].

Since both the cOA synthesis activity of Cas10-Csm and its detailed structure were only recently described, a fundamental description of how the complex senses bound target RNA to activate cOA synthesis is lacking [5,6,13,25]. To address this knowledge gap, we identified two residues in the Palm2 domain of Cas10 and three residues in domain 4 of Cas10 that line the target RNA binding channel and which we hypothesized would influence its interaction with target. We investigated how site-directed mutants of these residues affect interference in cells exposed to cognate and partially mismatched target RNAs. We purified a subset of these site-directed mutants and characterized their target RNA binding and cOA synthesis activity *in vitro*. We find both *in vivo* and *in vitro* that Cas10 residues lining the target RNA binding channel influence the specificity of interference.

## Materials and methods

### Docking of Cas10 into cryo-EM density

The AlphaFold2 model of *S. epidermidis* Cas10 given by AF-Q5HK89-F1-v3 was docked into the density given by EMD-27593 using real space refinement implemented in Phenix [26]. Each domain of Cas10 was fit as an independent rigid body. The PDB coordinates 8do6 give the atomic model of the Csm2-5 proteins, crRNA and target RNA for this map.

### Multiple sequence alignment

Cas10 sequence alignments were produced in the following manner: the AFDB clusters tool available at https://cluster.foldseek.com was queried with the sequence for *S. epidermidis* Cas10 (Q5HK89) returning the cluster A0A369ARA70 which contains 1080 Cas10 sequences [27]. The sequences were aligned with Clustal Omega and rendered as sequence logos with the WebLogo3 tool [28,29].

### Site-directed mutagenesis

The pACYC vector encoding *S. epidermidis* *CRISPR-Cas10* was a gift from Dr. Michael Terns of the University of Georgia [30]. The *CRISPR-Cas10* locus encodes a single spacer gene, *spc1*, which targets the *Nes* transcript, denoted pACYC-CRISPR-*spc1*. In a previous study, we developed a vector that replaced the *spc1* gene with *spc2*, resulting in pACYC-CRISPR-spc2 [31]. Both pACYC plasmids possess a chloramphenicol resistance marker. A map of pACYC-CRISPR-spc1 is given in Supplementary Data (Fig. S1) and plasmid sequences and all oligo and gene fragment sequences are also given (Table S1). For the site-directed mutants of pACYC-CRISPR-spc1, Q5 site-directed mutagenesis was used to introduce single, double, and triple mutations (New England Biolabs). For the quintuple mutant, a GeneArt string (ThermoFisher Scientific) was ordered containing the 5 mutations along *cas10*. Inverse PCR was used to linearize pACYC-CRISPR and Gibson Assembly was used to ligate the gene string into the vector. To create a pACYC-CRISPR-spc2 plasmid with *cas10* mutants, we began with the pACYC-CRISPR-spc1 plasmids carrying the *cas10* mutants. We utilized NcoI and PspXI restriction sites to remove *spc1* and DNA ligase to insert a GeneArt string (ThermoFisher Scientific) encoding *spc2*. All plasmids were verified by whole-plasmid sequencing.

A pTRC vector containing an ampicillin marker and a *nes* target gene (pTarget-nes) was also provided by Dr. Michael Terns (Fig. S2). We reported the insertion of a *cn20* target gene, into this plasmid to create pTarget-cn20 in a previous study (Fig. S3) [31]. In our current study, we constructed two new target plasmids: each has mismatches from +7 to + 12 along the crRNA-target RNA duplex and is denoted as MM2. Gibson assembly was used to introduce MM2 into pTarget-nes with a gene strand (Eurofins Genomics). For experiments targeting the *cn20* gene, Q5 PCR was used to introduce MM2. All constructs were verified by whole plasmid sequencing. The target plasmids denoted MM1 carrying mismatches in the crRNA-target duplex in positions +1 to + 6 were reported previously [31].

### Interference assays

*E. coli* BL21(DE3) cells containing pACYC-CRISPR wild type plasmids were made electrocompetent then transformed by electroporation with 100 ng of the corresponding pTarget plasmid. After transformation, the cells were resuspended with SOC medium and incubated at 37°C with shaking for 1 hour. Ten-fold serial dilutions were then prepared in LB medium, and the resulting suspensions were plated onto LB-agar plates containing either chloramphenicol (17 µg/mL) or a combination of chloramphenicol (17 µg/mL) and ampicillin (50 µg/mL). After overnight incubation at 37°C, the plates underwent quantification of colony-forming units per mL (CFU/mL) for three independent transformations of each target plasmid.

### Expression and purification of Cas10-Csm variants

One Shot BL21-AI (ThermoFisher Scientific) strains harbouring pACYC-CRISPR were grown in LB medium at 18°C with shaking until an OD_600_ of 0.8 was reached. The cultures were induced with 0.2% w/v L-arabinose and incubated at 26°C overnight. The cultures were then pelleted and stored at −20°C. On the day of purification, the pellets were resuspended in a solution of 50 mM NaH_2_PO_4_ pH 8.0, 300 mM NaCl, 10 mM imidazole, 0.1 mg/ml lysozyme, 1 mM PMSF, and 0.1% v/v Triton X-100. After incubating on ice for 1 hour, the cells were disrupted by sonication and cell debris was cleared by centrifugation. The lysate was then passed through a 0.8 µm filter. The clarified lysate was introduced to Ni-NTA resin, which was washed with 10 column volumes of wash buffer 1 (50 mM NaH_2_PO_4_ pH 8.0, 300 mM NaCl, 20 mM imidazole), followed by 10 column volumes of wash buffer 2 (50 mM NaH_2_PO_4_ pH 8.0, 300 mM NaCl, 20 mM imidazole, 10% v/v glycerol). Protein elution was performed using a stepwise increase of imidazole concentrations (100 mM and 250 mM) over ten column volumes. Fractions were analysed by SDS-PAGE to assess purity. Fractions containing the Cas10-Csm complex were combined and placed on a 5–20% (w/v) sucrose gradient in a solution of 50 mM Tris HCl pH 8.0, 150 mM NaCl, and 5% (v/v) glycerol. Ultracentrifugation was conducted for 41 hours at 31k rpm using a Beckman SW-32TI rotor. Fractions containing the Cas10-Csm complex were identified by A280. The purity of the samples was evaluated by a 4–20% SDS-PAGE gel. The final elutions were concentrated with a Pall centrifugal device with a 30 kDa MWCO membrane, flash-frozen, and stored at −80°C.

### crRNA extraction and validation

crRNAs were isolated from purified Cas10-Csm variants using extraction two times with phenol-chloroform-isoamyl alcohol (25:24:1) and one time with chloroform. The final aqueous layer was collected and a solution of 70% ice-cold ethanol and 300 mM of sodium acetate pH 5.2 was added. The sample was then incubated at −20°C overnight. The next day, the sample was centrifuged, and the supernatant was disposed of. The resulting pellet was rinsed with ethanol and dried down. The RNA was resuspended in water, and its concentration was measured using a Nanodrop spectrometer. To visualize the crRNA, the extracted RNA was radiolabelled using γ-32P-ATP, followed by separation on a 12% acrylamide urea-PAGE gel. The gels were exposed to a storage phosphor screen and imaged with a Typhoon FLA 7000 imager.

### Saturation affinity binding assays

RNA binding affinity was assessed using fluorescence polarization (FP). Four 43-nucleotide RNAs (Cognate-F, non-complementary-F, MM1-F, and MM2-F) based on the *Nes* target sequence, labelled with 5’ fluorescein and purified by PAGE were obtained from Horizon Discovery. Serial dilutions of Cas10-Csm with final concentrations from 1000–0.48 nM were combined with the fluorescein-labelled RNA and incubated for 2 hours at room temperature before FP was measured on a Bio-Tek Synergy H1 plate reader. The assay was performed in a buffer containing 50 mM Tris-HCl pH 8.0, 400 mM NH_4_Cl, 5% v/v glycerol, 0.025% w/v BSA, and 1 mM EDTA. Two replicate titrations were performed for each target RNA except the non-complementary negative control. No binding was evident for the 0.48 nM and 0.97 nM titration points, so the mean of these FP values was used to baseline correct the data. Data were fit by non-linear regression in GraphPad Prism 9.0 using the expression y = y_max × {R + x + Kd− [(R + x + Kd)^2^ −4Rx]^1/2^}/2 R. The fluorescein target RNA is *R* = 10 nM. Y_max was estimated from a fit of the 1000–62 nM titration points to a hyperbola. When K_d_ values are compared in the text, an F-test implemented in Prism 9.0 was used for ANOVA. For example, the K_d_ for cognate target RNA binding to wt complex was compared against the K_d_ for cognate binding to K524E complex or R754E complex.

### Target mismatch library

*In vitro* transcription was done using the Promega RiboMax kit with DNA templates obtained from Eurofins. DNA templates for cognate and +1 to + 11 mismatches (excluding +6 mismatch) were annealed and extended using Q5 DNA polymerase to create double stranded DNA. For a + 6 mismatch, two strands of DNA were combined, heated at 95°C for 5 min and then slowly cooled to room temperature to allow for annealing. Cleanup of the transcription products involved DNase I digestion, phenol-chloroform extraction with phenol-chloroform-isoamyl alcohol 25:24:1, and ethanol precipitation according to the manufacturer’s instructions. The final product was passed through a G-25 column to remove residual NTPs. The quality of the purified RNAs was verified by a 12% acrylamide urea-PAGE run at 150 V for 45 min, stained with SYBR green II, and imaged on a Typhoon FLA 7000 imager.

### COA synthesis assays

Three replicate reactions were performed in all cases. Reactions were performed with 100 nM Cas10-Csm and 400 nM target RNA in TNG buffer (50 mM Tris-HCl pH 8.0, 150 mM NH_4_Cl, 5% v/v glycerol), 500 μM ATP, 30 nM α-^32^P-ATP 3000 Ci/mmol, and 10 mM MgCl_2_ at 37°C for 1 hour. COA products were separated by thin-layer chromatography (TLC) using previously described methods [32]. Briefly, a chamber containing TLC running buffer, composed of 0.2 M ammonium bicarbonate pH 9.3, 70% ethanol, and 30% water was pre-warmed to 35°C to saturate the chamber with buffer vapour. Samples were spotted on a silica TLC plate 1.5 cm from the bottom of the plate and allowed to air-dry. The plate was placed in the chamber for 2 hrs, or until the solvent front was approximately 5 cm from the top. The plate was removed from the chamber, dried with a gentle air stream and cOAs were visualized by phosphorimaging on a Typhoon FLA 7000 imager. The two prominent cOA products were quantified by densitometry using ImageQuant software. A standard curve of serial dilutions of α-^32^P-ATP was also run on the TLC plate and subjected to phosphorimaging (Fig. S4). The standard curve was used to ensure our cOA products were within the linear range of the equipment and the slope extracted from linear regression of the curve was used to convert densitometry values to μM cOA (Figure S4). Log_2_ fold change was calculated by taking log_2_ of the relative amount of cOA synthesized in the presence of a mismatched target RNA divided by the amount synthesized with the cognate target RNA (*e.g*. log_2_ [cOA_K524E, +2_ /cOA_K524E, cognate_]). *p*-values were calculated with a two-tailed t-test for two samples of unequal variance. Uncropped images of the TLC plates are given in Figure S6.

### Steady-state kinetics

COA synthesis was monitored by detecting the release of pyrophosphate using a fluorescence-based Malachite Green Phosphate Assay Kit (MilliporeSigma). Reactions contained Cas10-Csm complex with a six-fold molar excess of RNA in buffer (50 mM Tris-HCl, pH 8.0; 150 mM NH_4_Cl; 5% [v/v] glycerol; 1 mM MgCl_2_; 50 to 1000 µM ATP; and 0.05 units Thermostable Inorganic Pyrophosphatase [TIPP, NEB M0296S]). Reactions were incubated at 37°C to initiate product formation. At various time points, aliquots were removed and heated at 75°C for 10 min to activate TIPP and quench complex activity. Absorbance at 620 nm was measured on a BioTek Synergy H1 plate reader. Phosphate concentrations were determined using a standard curve generated from the phosphate standards provided with the kit. Linear regression of phosphate concentration versus time was performed to determine initial velocities. Then data were fit to the model y=[E]_t_*k_cat_*x/(K_m_ + x) in Prism 9.0.

## Results

### Identifying Cas10 residues whose non-covalent interactions with target RNA may regulate interference

We sought to answer how interference would be affected by the alteration of semi-conserved to conserved Cas10 residues lining the target binding channel which likely engage in non-covalent interactions with target RNA. We believe answering this question will contribute to understanding the mechanistic basis for how cOA synthesis is activated upon target RNA binding to Cas10-Csm. Additionally, we believe this data will be relevant to generating designer Cas10 proteins for diagnostics that are either more tolerant or less tolerant to mismatches in the crRNA-target RNA duplex.

**Figure 2.**
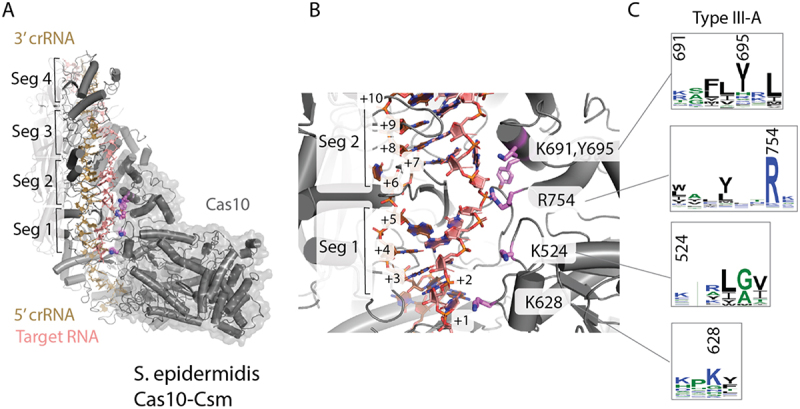
Cas10 in type III-A CRISPR systems contains basic residues and an aromatic residue in the target RNA binding channel poised to interact with the crRNA-target duplex. (A) The structure of *S. epidermidis* Cas10-Csm based on coordinates 8do6 (with an AlphaFold model of Cas10 docked to EM density) is shown. The crRNA-target duplex possesses four segments. Each segment contains five base-pairs and a flipped-out, unstacked nucleotide. (B) Five Cas10 residues poised to contact target RNA are shown as sticks. Cyclic oligoadenylate synthesis by Cas10 is sensitive to mispairing in segments 1 and 2. (C) The Cas10 residues of interest range from moderately to highly conserved in type III-A CRISPR systems.

We previously reported a cryo-EM reconstruction of *S. epidermidis* Cas10-Csm bound to an intact target RNA [11]. We used this model to identify Cas10 residues which may contact target RNA. The atomic model (PDB code 8do6) contains crRNA, target RNA and proteins Csm2-Csm5. Clear density for Cas10 exists, but an atomic model was not deposited due to a lack of clear side chain density. Coordinates for SeCas10-Csm bound to a cleaved target RNA have also been deposited in the PDB, however this structure has undergone substantial conformational changes compared to the structure bound to intact target [33]. We docked an AlphaFold2 model of Cas10 into the cryo-EM volume we previously reported (EMD-27953) and performed domain-wise rigid body fitting into it (Figure 2(A)). We selected Cas10 residues within 6 Å of the target RNA as those with potential non-covalent interactions to the target. The larger than usual distance, 6 Å, was used due to some uncertainty with regard to side chain positions. From the residues identified we noted four basic residues, K524, K628, K691 and R754 which seemed likely to interact with the target RNA (Figure 2(B,C)). An additional aromatic residue, Y695 from our list of potential interactors was also noted.

### A phenotypic assay to investigate the effect of Cas10 mutants on interference

**Figure 3.**
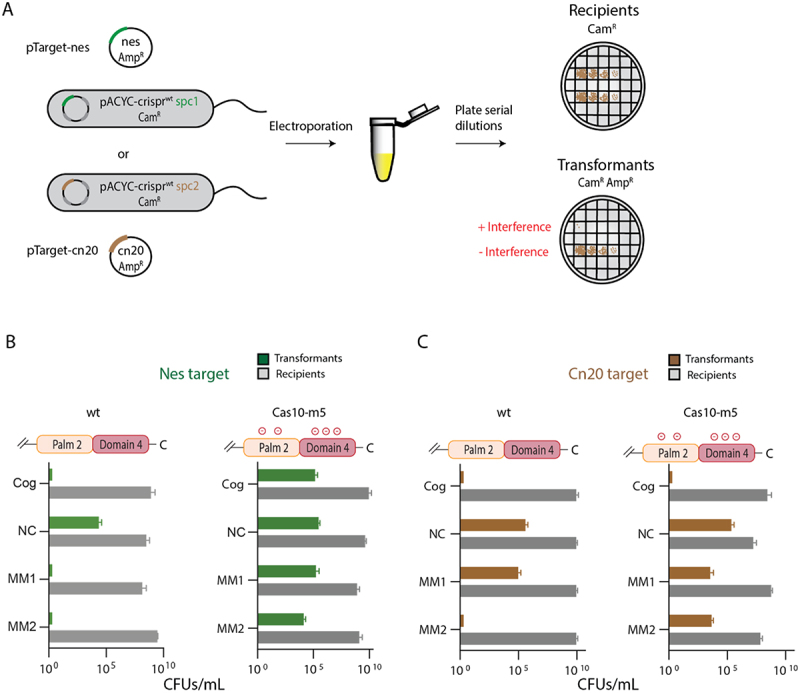
Cas10 residues in the target RNA binding channel regulate cOA-mediated interference. (A) Diagram of the cOA-mediated interference assay. *E. coli* cells harboring pACYC-CRISPR containing a spacer (*spc1*) that targets the *nes* gene are transformed with pTarget-nes and transformation efficiency reports on whether interference occurred. Alternatively, pACYC-CRISPR containing a spacer (*spc2*) that targets the *cn20* gene is used. Recipients are the electrocompetent cells capable of plasmid uptake and transformants are those that have taken up pTarget. (B) Cells harboring a wild type *cas10* interfere with a cognate (Cog) *nes* target sequence, a *nes* target in which segment 1 is mismatched (MM1) with crRNA and an *nes* target in which segment 2 is mismatched (MM2) with crRNA. Interference with a non-complementary target sequence (NC) is not observed and serves as a negative control. Cells harboring *cas10* with five target RNA binding channel residues mutated (K524E/K628E/K691E/Y695E/R754E: Cas10-m5) do not interfere with the *nes* targets assayed. (C) Cells harbouring wild type *cas10* interfere with a cognate *cn20* target sequence and a segment 2 mismatched target but not a segment 1 mismatched target. Cells harbouring Cas10-m5 do not interfere with either *cn20* target sequences containing a mismatched segment.

Several model systems have been reported which assay interference mediated by *S. epidermidis CRISPR-cas10*. This CRISPR-Cas system provides both anti-phage and anti-plasmid immunity. The latter has been assayed in *S. epidermidis* cells utilizing a conjugative plasmid. An extensive set of site-directed mutants and knockouts revealed which *CRISPR-cas10* components were necessary for anti-plasmid immunity specifically revealing that both the Palm2 domain of Cas10, which synthesizes cOA, and the Csm6 nuclease, which is activated by cOA, are required [34]. This demonstrates the crucial role of cOA in anti-plasmid immunity. *S. aureus* cells hosting *S. epidermidis CRISPR-cas10* on a plasmid have also been used to measure anti-plasmid immunity [35]. In these experiments a second plasmid encoding a transcript derived from the *cn20* phage gene, which is complementary to the *spc2* spacer, was used to transform the cells and transformation efficiency was measured. A third model system for anti-plasmid immunity uses *E. coli* hosting a plasmid expressing S. *epidermidis* Cas10-Csm which is then challenged by transformation with a plasmid containing a *nes* target sequence (complementary to *spc1*) or a *cn20* target sequence (complementary to *spc2*) (Figure 3(A)). The *E. coli* based system indicates the five proteins that make up the Cas10-Csm complex, a crRNA matured by Cas6 and the Csm6 nuclease are sufficient for anti-plasmid immunity [17].

We validated the *E. coli* based interference system with extensive controls. In type III CRISPR systems, the crRNA is composed of two sections, the 5’-tag and the body. A target RNA that base-pairs throughout the body section to the crRNA but not with the 5’-tag is termed a cognate target and elicits interference. As a positive control in our assay, *E. coli* expressing wild type Cas10-Csm were transformed with a plasmid encoding a cognate target RNA and as a negative control these cells were transformed with a plasmid encoding a non-complementary target RNA. In each case, the expected results were observed: no transformants were detected in the cognate case and approximately 10^5^ colony forming units per mL (CFU/mL) were observed in the non-complementary case (Figure 3(B)). *In vivo*, disruption of Watson-Crick base-pairing at several contiguous positions in the crRNA-target RNA duplex leads to a loss of interference [31,35,36]. We refer to these target sequences as mismatched. We generated target sequences that were mismatched at segment 1 (MM1) or segment 2 (MM2) and did this for both the *nes* transcript and the *cn20* transcript. When cells harbouring wild type *CRISPR-cas10* were challenged with the mismatched target sequences, interference followed a pattern that is consistent with previous data [31]. That is, interference was unaffected by the mismatches in the *nes* sequence context (Figure 3(B)). However, in the *cn20* sequence context MM1 caused a loss of interference but MM2 did not (Figure 3(C)). These results confirm previous reports that when the effects of mismatched nucleotides on interference are assayed the identity of the mismatched and the surrounding nucleotides matter [31]. We speculate that the G/C content of each segment may drive this phenomenon: G/C rich segments of the crRNA-target RNA duplex better promote the conformational changes in Cas10 required for cOA synthesis.

### Cas10 residues contacting target RNA regulate whether mismatched targets activate interference

We created a site-directed mutant of *cas10* altered at the five positions described above: K524E, K628E, K691E, Y695E and R754E. We refer to the construct possessing these mutants as Cas10-m5. Glu residues were introduced with the expectation that insertion of a negative charge adjacent to the phosphodiester backbone of target RNA would be unfavourable and may produce a potent phenotype. We utilized the *E. coli* based anti-plasmid immunity system to assay the effect of Cas10-m5 on interference. When transformed with a *nes* target sequence, Cas10-m5 is defective in interference for all versions of the target where interference could be expected: cognate, MM1 and MM2 target sequences (Figure 3(B)). When transformed with a *cn20* target sequence, Cas10-m5 supports interference on a cognate target sequence but not MM1 or MM2 (Figure 3(C)). Therefore, in the *cn20* context, the mutant residues appear to sensitize Cas10-Csm to mismatches in segment 2 (Figure 2(A)). In the *nes* context, the mutant residues lead to a loss of interference for all versions of this target sequence.

**Figure 4.**
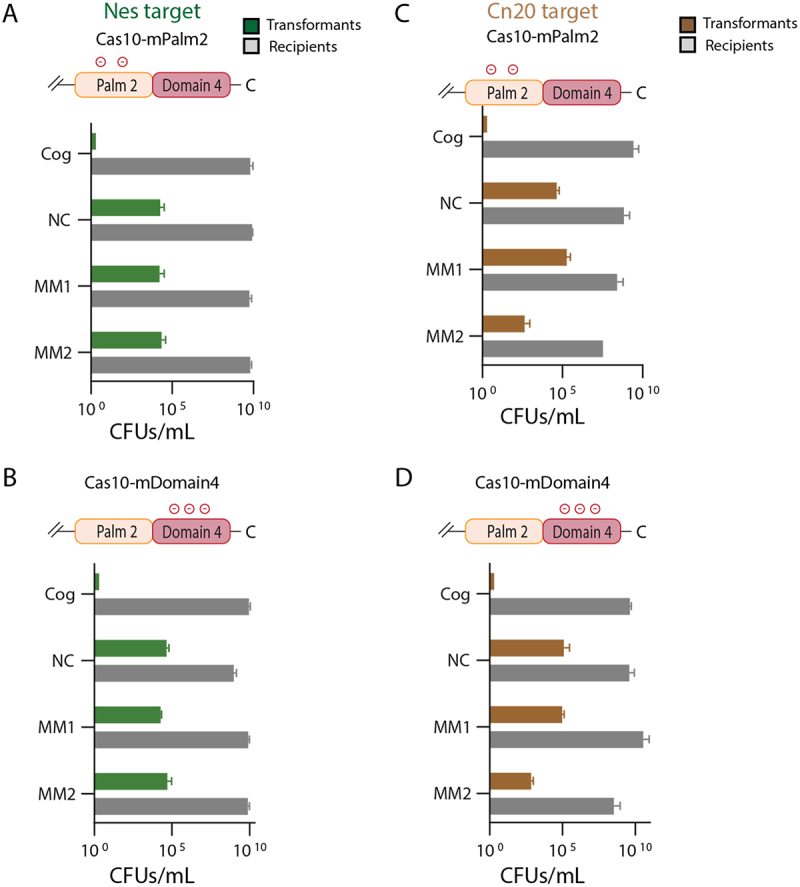
Palm2 multi-mutants and domain 4 multi-mutants retain interference against cognate target sequences but not mismatched targets. (A) Cells harboring the K524E/K628E Palm2 mutant (Cas10-mPalm2) maintain interference against a cognate (Cog) *nes* target but lose interference if segment 1 is mismatched to crRNA (MM1) or if segment 2 is mismatched to crRNA (MM2). A negative control, non-complementary target is indicated by NC. (B) Cells harboring the K691E/Y695E/R754E domain 4 mutant (Cas10-mDomain4) were assayed against the panel of targets. (C) and (D) Cas10-mPalm2 and Cas10-mDomain4 were assayed for interference against a panel of *cn20* targets.

The K524 and K628 residues are located in the Palm2 domain of Cas10 while K691, Y695 and R754 are located in domain 4. To score the relative importance of the two domains in contributing to interference, we next performed interference assays with constructs containing only the two Palm2 mutants (Cas10-mPalm2) or the three domain 4 mutants (Cas10-mDomain4). In the *nes* context, interference was restored in the presence of cognate target sequence for both Cas10-mPalm2 and Cas10-mDomain4 (Figure 4(A,B)). Therefore, both mutants were more capable of interference than Cas10-m5. In the *cn20* context, the interference phenotype of Cas10-mPalm2 and Cas10-mDomain4 was similar to Cas10-m5 (Figure 4(C,D)). Interference occurred on the cognate target sequence but was defective on the mismatched target sequences. The behaviour with the MM1 target sequence was expected. Since wild type is defective in interference with MM1 target sequences these assays function as a negative control. Notably, transformation efficiency was lower for the MM2 target sequence in these experiments (10^3^ for both Cas10-mPalm2 and Cas10-mDomain4 versus 10^4^ for Cas10-m5) than in the corresponding Cas10-m5 experiment, indicating Cas10-m5 is slightly more defective in interference in this context. In summary, the defects in interference in Cas10-m5 can’t be assigned solely to site-directed mutants of Palm2 or domain 4 but instead both contribute to the property.

**Figure 5.**
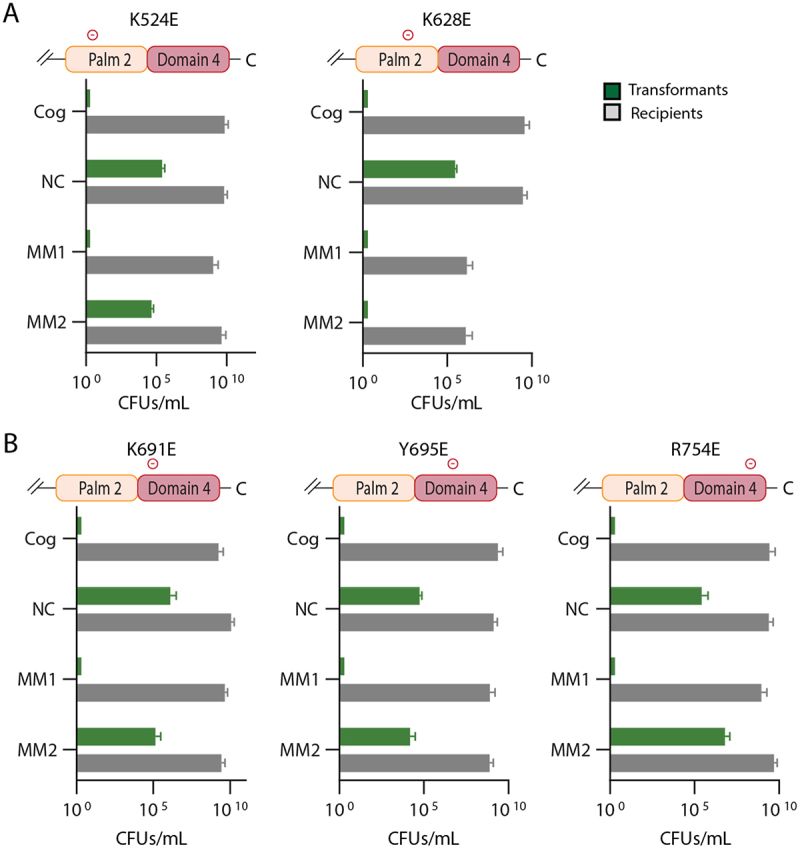
Single site-directed mutants of Palm2 or domain 4 possess interference defects with segment 2 mismatched target sequences but not with cognate sequences. (A) The K524E and K628E site-directed mutants of Palm2 were assayed. (B) the K691E, Y695E and R754E site-directed mutants of domain 4 were assayed. In all cases a *nes* target was used.

We next assayed the contribution to interference of single site-directed mutants of each of the chosen residues. In the *nes* context, K628E supports interference on cognate, MM1 and MM2 target sequences behaving identically to wild type (Figure 5(A)). Interestingly, K524E, K691E, Y695E and R754E support interference with both cognate and MM1 target sequences but are defective on MM2 targets (Figure 5(A,B)). These results suggest the four mutant residues each sensitize Cas10-Csm to mismatches in segment 2: while wild type Cas10-Csm possesses a normal interference phenotype on an *Nes* target with MM2, the four single site-directed mutants lose interference. Therefore, the four residues are candidates for a designer Cas10 with enhanced discrimination of mismatches.

**Figure 6.**
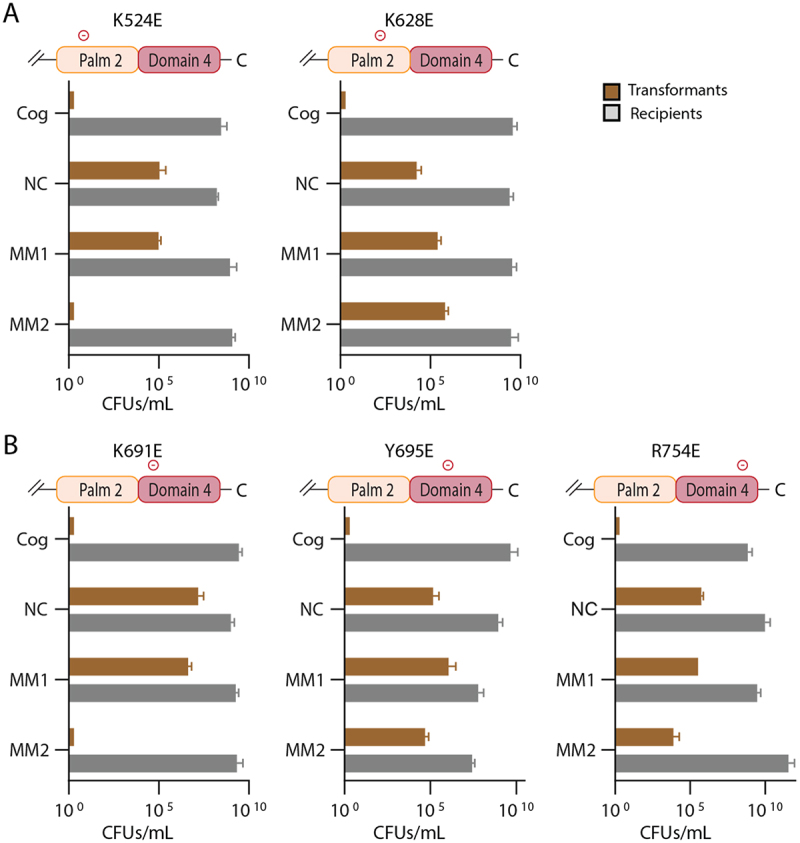
Single site-directed mutants of Palm2 or domain 4 possess interference defects with segment 2 mismatched target sequences but not with cognate sequences. (A) The K524E and K628E site-directed mutants of Palm2 were assayed. (B) the K691E, Y695E and R754E site-directed mutants of domain 4 were assayed. In all cases a *cn20* target was used.

**Figure 7.**
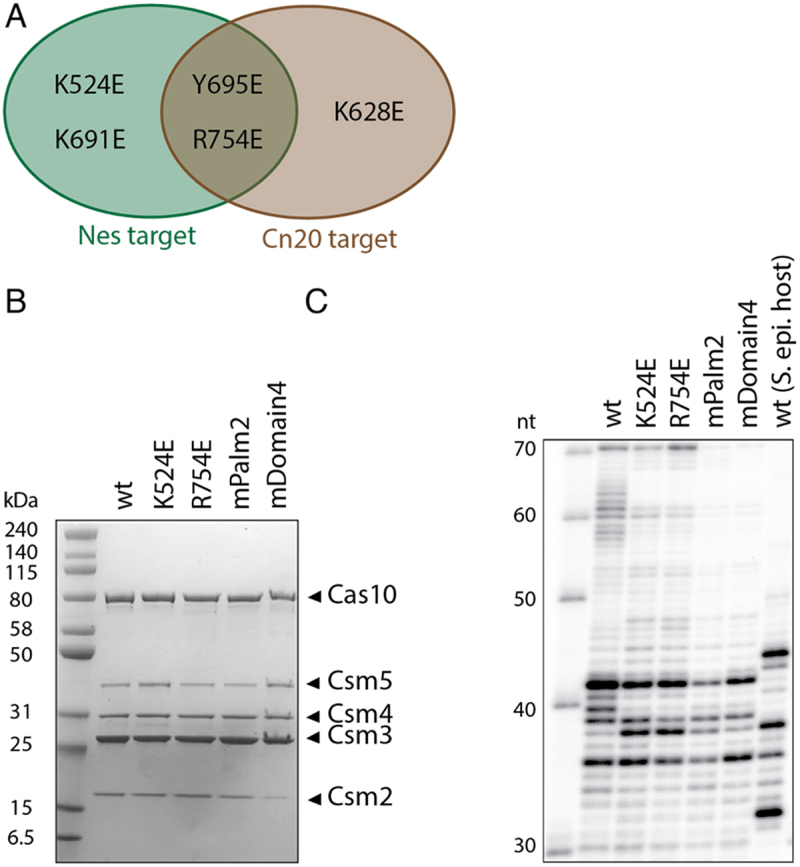
A Venn diagram indicates the scenarios where Cas10 mutants induce sensitivity to mismatches and a PAGE analysis of Cas10-Csm complexes is given. (A) A Venn diagram indicates that Cas10 mutants Y695E and R754E display heightened sensitivity to mismatches in our in vivo interference assay for both *Nes* and *Cn20* target sequences. (B) SDS-PAGE of site-directed mutants reveals all five Cas10-Csm proteins are present in the purified complexes, however the mDomain4 mutant has diminished Csm2. (C) Urea-PAGE of RNA extracted from site-directed mutants indicates these complexes possess mostly mature crRNA rather than unprocessed, pre-crRNA (length 70 nts). However, the mPalm2 complex is depleted in mature crRNA.

We performed the same assay, measuring interference with single site-directed mutants of *cas10*, but now in the *cn20* sequence context. As noted before, MM1 target sequences function as a negative control because wild type is deficient in interference with these targets (Figure 3(C)). In these experiments, K524E and K691E have interference phenotypes similar to wild type: interference occurs for cognate and MM2 target sequences. In contrast K628E, Y695E and R754E have a loss of interference for the MM2 target sequence (Figure 6(A,B)). Considering all the data for single site-directed mutants reveals that K524E, K628E and K691E appear to sensitize Cas10-Csm to mismatches in a sequence context–dependent manner while Y695E and R754E sensitize Cas10-Csm to mismatches in both sequence contexts tested (Figure 7(A)).

### Purification of mutant Cas10-Csm complexes

Next, we performed *in vitro* assays to gain mechanistic insights. We sought to answer to what degree decreased interference on mismatched target sequences was due to altered activity versus defects in expression and assembly. We examined how the mutants K524E, R754E, Cas10-mPalm2 and Cas10-mDomain4 affected behaviour *in vitro*. These four complexes were chosen for two reasons. First, they all support interference *in* *vivo* on cognate target sequences, suggesting they may be capable of cOA synthesis *in vitro*. Second, they are expected to span a range of potential behaviours. For example, K524E sensitizes Cas10-Csm to mismatches in the *Nes* sequence context while R754E sensitizes the complex to mismatches in both sequence contexts tested.

First, we examined whether the four Cas10-Csm mutants could be purified as intact ribonucleoprotein complexes. PAGE analysis indicated all five proteins that comprise the complex are present, although Csm2 appears to be present at sub-stoichiometric levels in mDomain4 (Figure 7(B)). We also performed a PAGE analysis of the crRNA content of the mutants. Wild type complex has multiple bands in the range of 30–70 nt with 42 nt being most prominent (Figure 7(C)). The 5’ end of crRNA is matured by the Cas6 nuclease which is present on the plasmid we use for recombinant expression (Fig. S1). The 3’ end of the crRNA is matured by the PNPase and RNase R nucleases which both physically interact with Csm5 to coordinate this activity [37,38]. Since both PNPase and RNase R are present in *E. coli*, normal maturation of crRNA is expected. We observe a different distribution of crRNA sizes during recombinant expression than has been observed when Cas10-Csm is purified from native *S. epidermidis* cells [39]. Nevertheless, the most abundant band from recombinant expression, 42 nt, is similar to the most abundant bands, 37 nt and 43 nt, observed when SeCas10-Csm is purified from *S. epidermidis* (Figure 7(C)). The four mutant Cas10-Csm complexes possess crRNA bands with lengths similar to wild type (Figure 7(C)). Analysis of mPalm2 and mDomain4 crRNA suggest they are approximately 41% and 67% respectively as abundant as wild type (Fig. S5). We concluded at this point that the single Cas10 mutants efficiently mature into active Cas10-Csm complexes while the multi-mutants mature with moderate efficiency. We focused the remainder of our *in vitro* analysis on the single mutants.

### Target RNA affinity for Cas10 K524E or R754E

**Figure 8.**
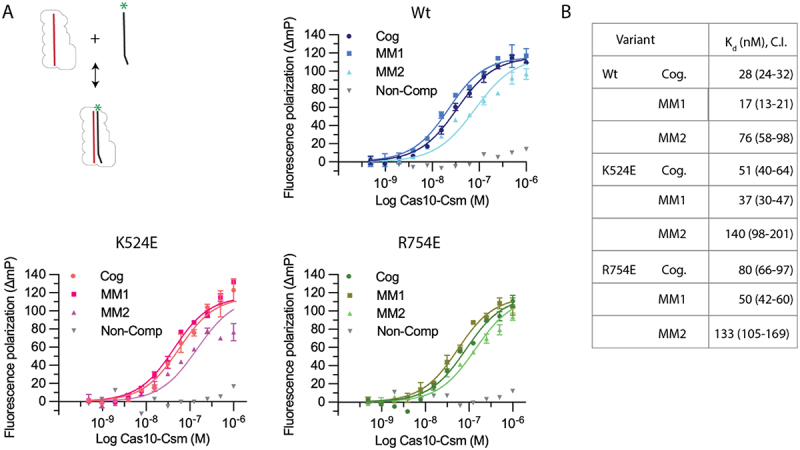
The affinity of *Nes* target RNA binding to Cas10-Csm variants K524E and R754E. (A) Fluorescently labelled target RNAs were incubated with wild type Cas10-Csm and variants. Binding affinity was measured by fluorescence polarization. Target RNA sequences used are cognate (Cog), a target with segment 1 mismatched (MM1), a target with segment 2 mismatched (MM2) and a non-complementary, negative control target (Non-comp). Two replicate titrations were performed for each target RNA (except the negative control) and best-fit curves were determined by non-linear regression. Mean and standard error are plotted for each point in the titration. (B) Dissociation constant (K_d_) values for Cas10-Csm binding to target RNAs. Parentheses indicate 95% confidence interval.

Next, we sought to determine whether the K524E and R754E site-directed mutants had normal or altered target RNA binding and importantly how affinity was affected by segment mismatches, MM1 and MM2. To quantitate the effect of the mutants on target RNA binding we performed an affinity binding assay with a fluorescently labelled *Nes* cognate target RNA and labelled mismatch *Nes* target RNAs (Figure 8(A)). Experiments were performed in the absence of [Mg^2+^] to prohibit target RNA cleavage by the Csm3 component of the complex. Wild type binding to a cognate target RNA served as a positive control and binding to a fluorescently labelled non-complementary RNA (scrambled sequence) served as a negative control. The fraction active for both K524E and R754E was estimated to be approximately equal to wild type. A comparison of the K_d_ values of cognate target RNA for wild type complex compared to K524E and R754E reveals there is a significant difference for the mutants (*p* < 0.001 in both cases). They bind 1.8- and 2.8-fold weaker respectively (Figure 8(B)). However, our main interest was in whether the mutants could still bind mismatched target RNAs with reasonable affinity. Compared to cognate target RNAs, K524E and R754E K_d_ values were lower for the MM1 target indicating tighter binding (*p* < 0.01 and *p* < 0.001 respectively) and were only mildly higher for the MM2 target RNA (*p* < 0.001 in both cases), 2.7- and 1.6-fold respectively. In sum these site-directed mutants cause only modest alterations in target RNA binding affinity.

### Cas10 K524E or R754E regulate whether mismatched target RNAs activate cOA synthesis *in vitro*

**Figure 9.**
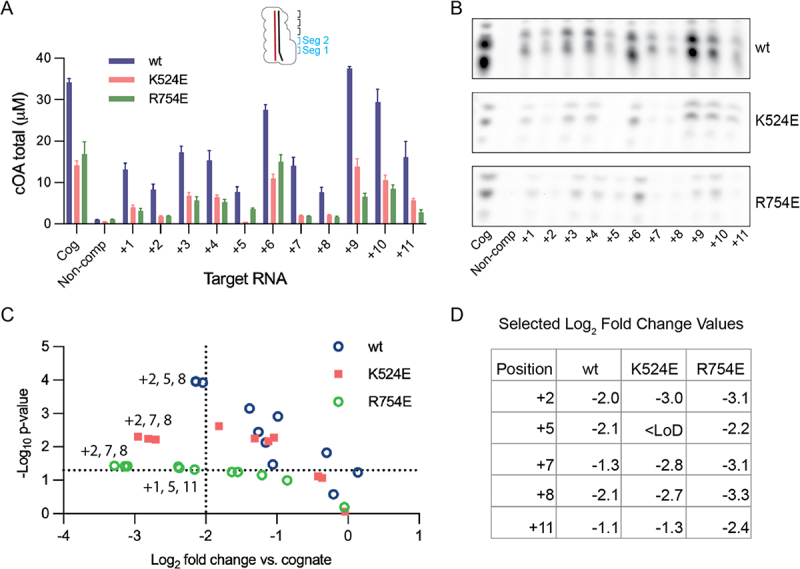
Cas10 mutants enhance sensitivity to single mismatches during *in vitro* cOA synthesis. (A) Target RNA based on the *Nes* sequence with single mismatches at positions +1 through +11 was used to stimulate cOA synthesis by Cas10-Csm. The amount of cOA produced from three replicate reactions was quantified. The mean and standard error of the mean are plotted. COA amounts for non-complementary target RNA and the K524E variant stimulated by the +5 target RNA were below the limit of detection and are plotted at that limit, y = 1.0 μM. (B) Images of representative TLC plates used for cOA quantitation. (C) A volcano plot of the data in panel a is shown. The amount of cOA synthesized by each Cas10 variant when bound to a single mismatch target RNA is compared to the cOA amount when a cognate target is used. The K524E + 5 data point is not plotted because it is below our limit of detection (LoD). Data points with *p* < 0.05 and at least a 4-fold decrease compared to cognate target RNA are labelled. (D) A table of selected log_2_ fold change values is given.

Having confirmed K524E and R754E were competent in target RNA binding, we sought to determine how cOA synthesis may be altered. We hypothesized that cOA synthesis by Cas10 site-directed mutants may be more sensitive to mismatches in the crRNA-target duplex than wild type Cas10-Csm. To test this, we *in vitro* transcribed a panel of *Nes* target RNAs containing either cognate sequence or a single mismatch in positions +1 to + 11 of the duplex, which comprise segments 1 and 2. This is similar to boundaries of the crRNA-target duplex described as the Cas10 activating region [21]. We performed cOA synthesis reactions *in vitro* with purified Cas10-Csm, a member of the target RNA panel and α-^32^P-ATP. The radioactive cOA products were separated by TLC and quantitated (Figure 9(A,B)). It has previously been shown that when wild type SeCas10-Csm is presented an *Nes* target RNA with mismatches in either position +2, +5, +7, +8 or + 11 a substantial reduction of cOA products occurs [16]. We observed that for the K524E and R754E mutants this phenomenon is enhanced. We generated a volcano plot to visualize the statistical significance derived from three replicate reactions plotted against the fold change (Figure 9(C)). Notably, fold change is a comparison of the cOA synthesis reaction using a mismatched target RNA to the reaction for the same Cas10 variant but in the presence of a cognate target. Dashed lines highlight the reactions with *p* < 0.05 that also have pronounced defects in cOA synthesis. Since we have plotted fold change versus cognate target RNA, the reactions left of the vertical dashed line demonstrate that for specific mismatches the K524E and R754E mutants possess enhanced discrimination against mismatched targets. That is, K524E and R754E possess 42% and 49%, respectively, of the activity on a cognate target RNA exhibited by wild type but have an outsize defect in cOA synthesis on several mismatches in the Cas10 activating region. The positions of greatest interest are labelled in Figure 9(C).

### k_cat_ for cOA synthesis is affected by a + 7 mismatched target RNA or the R754E mutant

**Figure 10.**
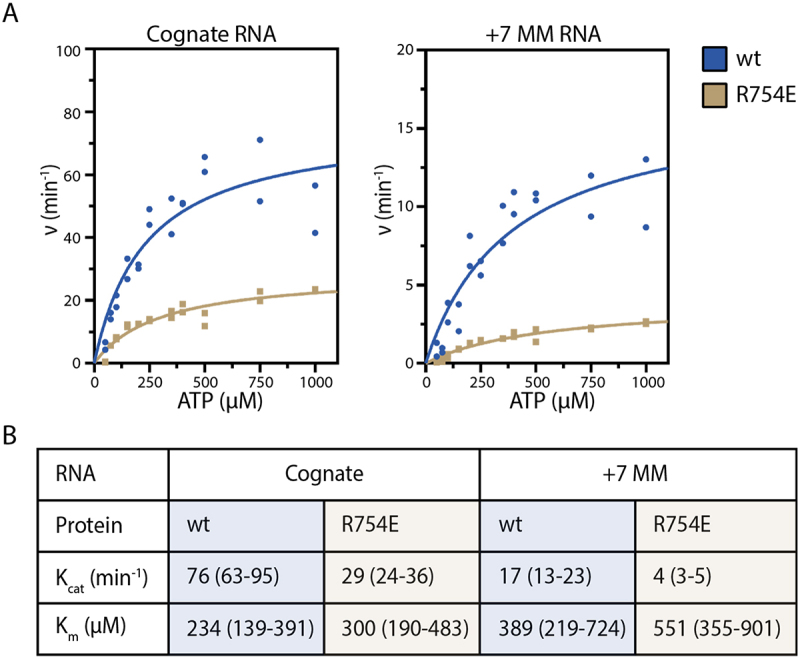
Steady-state kinetics for cOA synthesis by wt and R754E Cas10-Csm. (A) Formation of the pyrophosphate product of cOA synthesis was followed and data were fit to the Michaelis-Menten model. (B) K_m_ and k_cat_ parameters derived from non-linear regression of the data in (A) are shown with the 95% confidence interval.

To gain mechanistic insight into how target RNA mismatches or Cas10 mutants may affect cOA synthesis we performed steady-state kinetics (Figure 10(A)). Two plausible models for the decrease in cOA synthesis seen in Figure 9 are either target RNA mismatches or a Cas10 mutant cause a change in K_m_ for ATP or that a change in k_cat_ for the reaction occurs. Since the mutants and target RNA are located approximately 30 Å from the active site the effects must be mediated allosterically by influencing a conformational change. A K_m_ effect should then be interpreted as influencing a conformational change that drives ATP binding while a k_cat_ effect as a conformational change that positions the two ATP molecules to promote chemistry. When comparing wt to R754E in the presence of a cognate target RNA, a modest K_m_ difference is seen that is not statistically significant (Figure 10(B)). However, a k_cat_ difference is observed of 2.6-fold. When comparing wt bound to cognate or + 7 mismatch target RNA, again a modest K_m_ effect is observed that is not statistically significant but a 4.5-fold affect is seen in k_cat_ (Figure 10(B)). These data are consistent with a model in which some mismatches in the Cas10-activating region of target RNA and some Cas10 contacts to target RNA regulate cOA synthesis by promoting a conformational change required for catalysis, most likely positioning the ATP substrate close to the growing oligoadenylate chain. Importantly, when we compare how k_cat_ changes for wt Cas10-Csm on the +7 mismatch versus R754E on the mismatched target RNA we see results consistent with Figure 9: the k_cat_ change for R754E is larger.

## Discussion

Proper licencing of interference in CRISPR-Cas systems is important for the fitness of prokaryotes. Overly strict base-pairing requirements between crRNA and target RNA could lead to the failure of a bacterium to activate interference against a virus that accrued subtle sequence variations since spacer acquisition. Conversely, loose base-pairing requirements could lead to interference being activated by native prokaryotic genes. For type III-A CRISPR systems the mechanisms of licencing have not been fully described. We performed *in vivo* and *in vitro* experiments to determine whether residues lining the target RNA binding channel contribute to Cas10-Csm’s ability to distinguish cognate from mismatched targets and found that they do. Both *in vivo* and *in vitro* experiments support this conclusion. When assayed *in* *vivo*, the site-directed mutants K524E, K628E and K691E had context-specific effects on target sequence discrimination. By contrast, Y695E and R754E enhanced target sequence discrimination *in* *vivo* for both sequences, *Nes* and *Cn20*, we tested. *In vitro* experiments further supported our finding: K524E and particularly R754E displayed enhanced discrimination for some single mismatches between crRNA and target RNA. The *in vitro* result has bearing on the design and utility of nucleic acid diagnostics based on the Cas10 CRISPR system.

Comparison among our *in* *vivo* and *in vitro* data reveals broad agreement; however, certain specifics require explanation. The mPalm2 and mDomain4 multi-mutants display robust interference against a cognate target RNA *in* *vivo*, but *in vitro* mPalm2 cOA synthesis is 20% of cognate and mDomain4 synthesizes so little cOA that it is below our limit of detection (Fig. S7). There are several possible explanations for this. First, we see evidence of reduced stability of purified mPalm2 due to its lower amount of bound crRNA and for mDomain4 its lower level of bound crRNA and its reduced amount of bound Csm2. Another relevant factor is that the level of cOA required for interference *in* *vivo* may be very low and *in* *vivo* phenomenon such as product inhibition are not in play: in cells cOA are sequestered by binding to CARF domain containing proteins after synthesis while *in vitro* they are not sequestered and can potentially participate in product inhibition of Cas10. We also note that segment mismatches between crRNA and target RNA were used in our *in* *vivo* assay and that multiple mismatches are required in these assays to observe an interference phenotype. However, in our *in vitro* cOA synthesis assays, single mismatches between the crRNA and target RNA are observed to be capable of dramatically reducing cOA production. These phenomena have been observed by us and others [10,16,21,22,31,35,36,40]. It has been shown that the complementarity between the crRNA and target RNA required for interference *in* *vivo* is affected by target abundance [40]. Therefore, the discrepancy between the complementarity needed for *in* *vivo* interference versus *in vitro* cOA synthesis may be the result of the fact that our interference assay uses a high copy number plasmid driven by a T7 promoter resulting in high expression of the target RNA.

Due to their utility in gene editing, engineering CRISPR-Cas systems for enhanced specificity has been an area of major interest. A comparison of our work with experiments to engineer high fidelity variants of Cas9 points to future tactics that could be used. Early experiments showed that pruning *S. pyogenes* Cas9 contacts to either the target DNA strand or the non-target DNA strand reduced cleavage of off-target sequenced an approach similar to what we have taken with Cas10 [41,42]. Later single molecule and time resolved cryo-EM experiments of SpCas9 bound to cognate and mismatched targets revealed the importance of multiple checkpoints and concerted conformational changes that proceed DNA cleavage [43,44]. The HNH domain of SpCas9 is dynamic until the complex binds a cognate DNA which promotes formation of the active conformation of HNH which in turn allosterically activates the RuvC nuclease domain for concerted strand cleavage [43]. Two loops, L1 and L2, that link the HNH domain and the RuvC domain interact with the minor groove of the guide RNA-target DNA heteroduplex and are critical for activating SpCas9 along with of kinking of the heteroduplex [44]. Recognition of the role of these events in specificity and structures of SpCas9 bound to mismatched targets has led to new approaches for engineering SpCas9 specificity [44,45]. It has been demonstrated that Cas10-Csm undergoes significant conformational changes in Csm2 and Cas10 upon target RNA binding [11,13,14]. A clearer understanding of how these conformational changes specifically promote Cas10 activation may open new avenues for engineering Cas10.

The realization that CRISPR-Cas systems are well-suited to serve as molecular diagnostics was realized early in the history of CRISPR biotechnology [46,47]. CRISPR-Cas systems have been harnessed to detect RNA viruses, DNA viruses and human single nucleotide polymorphisms [47–50]. Each CRISPR-Cas system has intrinsic strengths and limitations as a diagnostic tool. For example, as an RNA targeting system, Cas10 is well-suited for detection of RNA viruses and has been deployed to sensitively detect SARS-CoV-2 RNA [20–23]. A strength for Cas10-Csm is its ability for signal amplification: each target RNA detection event initiates a multiple turnover reaction producing cOA which then activate a CARF domain containing enzyme, such as Csm6, which also performs a multi-turnover reaction such as collateral RNA cleavage. A limitation of Cas10-Csm is its loose specificity. Our data show that rational mutagenesis can improve the specificity of Cas10-Csm in two sequence contexts. Additional experiments will be needed to know if this is true across a broader variety of target RNA sequences.

## Supplementary Material

Khweis_et_al_SM_7.docx

## Data Availability

The authors confirm that the data supporting the findings of this study are available within the article [and/or] its supplementary materials.

## Pre-print statement

A pre-print version of this manuscript is available on bioRxiv (https://www.biorxiv.org/content/10.1101/2025.04.15.649021v1) [51].
