# Supplementary material for: Cas10 residues lining the target RNA binding channel regulate interference by distinguishing cognate target RNA from mismatched targets
Source: RNA Biol. 2026 Feb 18;23(1):1–18. doi: 10.1080/15476286.2026.2633385 (PMC12934148; doi:10.1080/15476286.2026.2633385)
Supplement: Khweis_et_al_SM_7.docx [file KRNB_A_2633385_SM1848.docx]

Cas10 residues lining the target RNA binding channel regulate interference by distinguishing cognate target RNA from mismatched targets

Sarah A. Khweis^1^, Mason A. Blackburn^1^, Calvin C. Perdigao^1^, Megan O. Pierce^1^, Colby R. Lewis^1^ and Jack A. Dunkle^1^*

^1^ Department of Chemistry and Biochemistry, University of Alabama, Tuscaloosa, AL, USA

*Corresponding author: Jack A. Dunkle

E-mail: [jadunkle@ua.edu](mailto:jadunkle@ua.edu)

**Supplemental Material**

**
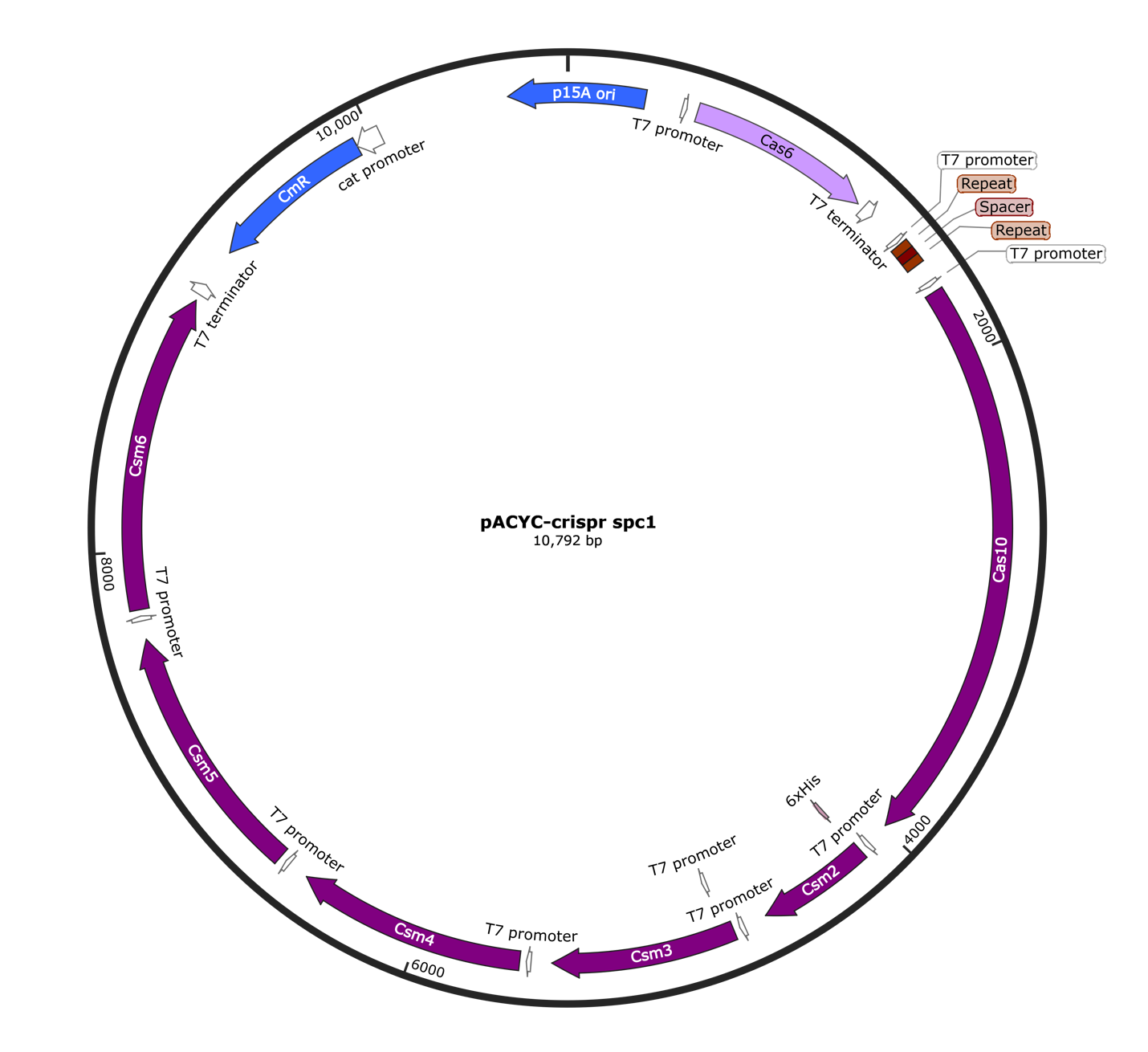
**

**Figure S1. The map pACYC-CRISPR-spc1.**


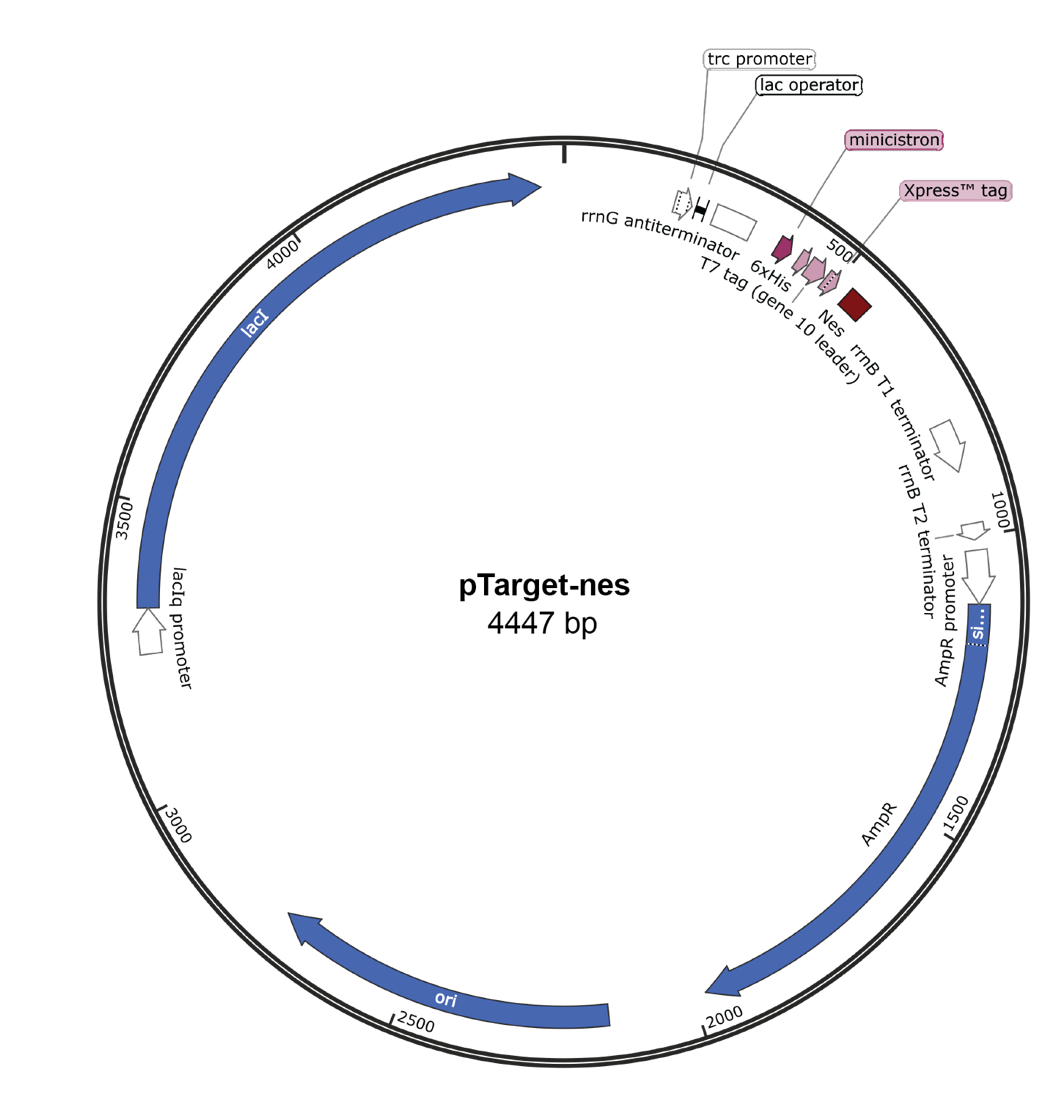


**Figure S2. The map of pTarget-nes.**


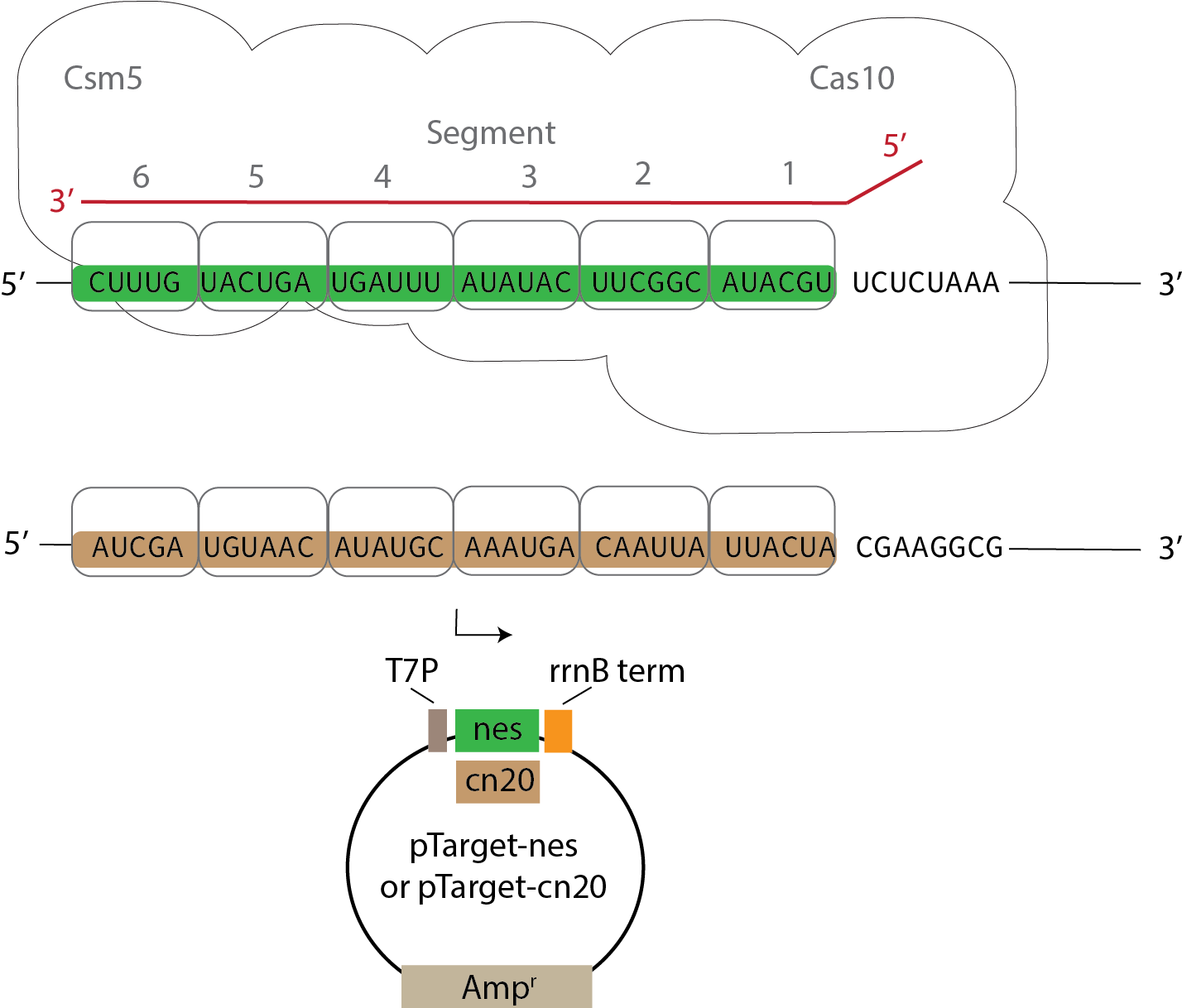


**Figure S3. A schematic of the transcripts encoded by pTarget-nes and pTarget-cn20.**

**Table S1. Plasmid sequences, gene fragments and oligos used in the study.**

| **Name** | **Sequence** | **Description** |
| --- | --- | --- |
| pr_K524E_F | GCGTCTGGGTGTTGTTCGTGCA | Fwd primer to introduce K524E mutation into cas10 |
| pr_K524E_R | TCAACACCTTCTTCACGATCAACATAG | Rev primer to introduce K524E mutation into cas10 |
| pr_K628E_F | GTATCCGGTTAGCAAAATGGCC | Fwd primer to introduce K628E mutation into cas10 |
| pr_K628E_R | TCACCGCTAAACATGCCAACACC | Rev primer to introduce K628E mutation into cas10 |
| pr_K691E_F | GGCCTTCATTTACAAAATGCTG | Fwd primer to introduce K691E mutation into cas10 |
| pr_K691E_R | TCACCATGTTCATCGGTCTGGCT | Rev primer to introduce K691E mutation into cas10 |
| pr_MAB_017 | AGCCTTCATTGAAAAAATGCTGG | Fwd primer to introduce Y695E mutation into cas10 |
| pr_MAB_018 | TTACCATGTTCATCGG | Rev primer to introduce Y695E mutation into cas10 |
| pr_K691EY695E_F | CATTGAGAAAATGCTGGCACTGCTG | Fwd primer to introduce K691E and Y695E mutations into cas10 |
| pr_K691EY695E_R | AAGGCCTCACCATGTTCATCGGTCTG | Rev primer to introduce K691E and Y695E mutations into cas10 |
| pr_R754E_F | GGAAGCCGACTAACATATGGCT | Fwd primer to introduce R754E mutation into cas10 |
| pr_R754E_R | TCGATCTGATAGATATAGTATTCCAGGG | Rev primer to introduce R754E mutation into cas10 |
| pACYC_Cas10_5x | CAGAATAATAACCAGGTTCGCATCTATAGCAAAAACAAACCGTATATTGGCATTGGCATTAGCACCAATCTGTGGATGTGTGATTATGATTATGCAAGCCAGAATCAGGATATGCGCGAAAAAGGTATTGGTAGCTATGTTGATCGTGAAGAAGGTGTTGAGCGTCTGGGTGTTGTTCGTGCAGATATTGATAATCTGGGTGCAACCTTTATTAGCGGCATTCCGGAAAAATACAATAGCATTAGCCGTACCGCAACCCTGAGCCGTCAGCTGAGTCTGTTCTTTAAATACGAGCTGAACCATCTGCTGGAAAACTATCAGATTACCGCAATTTATAGTGGCGGAGATGACCTGTTTCTGATTGGTGCATGGGATGATATTATCGAAGCGAGCATTTACATCAACGATAAATTCAAAGAGTTTACCCTGGACAAACTGACCCTGAGTGCCGGTGTTGGCATGTTTAGCGGTGAGTATCCGGTTAGCAAAATGGCCTTTGAGACAGGTCGTCTGGAAGAGGCAGCAAAAACTGGCGAAAAAAACCAGATTAGTCTGTGGCTGCAAGAGAAAGTGTATAACTGGGATGAGTTCAAAAAAAACATTCTGGAAGAGAAACTGCTGGTTCTGCAGCAGGGTTTTAGCCAGACCGATGAACATGGTGAGGCCTTCATTGAGAAAATGCTGGCACTGCTGCGTAATAACGAAGCAATTAACATTGCACGTCTGGCATACCTGCTGGCACGTAGTAAAATGAATGAAGATTTCACCAGCAAAATCTTTAACTGGGCACAGAACGACAAAGACAAAAATCAACTGATTACAGCCCTGGAATACTATATCTATCAGATCGAAGAAGCCGACTAACATATGGCTGCGTGG | Gene segment encoding Cas10-m5 quintuple mutant |
| Spc2 gene segment | CACTATAGGGAGACCATGGGATCGATACCCACCCCGAAGAAAAGGGGACGAGAACTAGTAATAATTGTCATTTGCATACGTTACATCGATGATCGATACCCACCCCGAAGAAAAGGGGACGAGAACCTCGAGGCTGTGGTCTAGACATTC | ThermoFisher GeneArt string used to replace spc1 in pACYC-CRISPR-spc1 with spc2 |
| Cn20 gene segment | GGATCTGTACGACGATGACGATAAGGATCCAACCCTTTTCCAAGCTTATCGATGTAACATATGCAAATGACAATTATTACTACGAAGGCGGCATGCTTCCAAGGCGAATTCGAAGCTTGGCTGTTTTGGCGGATGAGAGAAGATTTTCAGCCTGATACAGATTAAATCAGAAC | Gene segment to replace nes in pTarget to encode a cn20 target. |
| pTRC_nes_MM2 | GGGATCTGTACGACGATGACGATAAGGATCCAACCCTTTTCCAAGCTTCTTTGTACTGATGATTTATATACAAGCCGATACGTCAAGAGCAGCATGCTTCCAAGGCGAATTCGAAGCTTGGCTGTTTTGGCGGATGAGAGAAGATTTTCAGCCTGATACAGATTAAATCA | Gene segment to clone MM2 from +7 to +12 into pTarget-nes |
| pr_SAK_011 | GCCTGATACAGATTAAATCAGAAC | Primer to linearize pTRC to excise spacer coding region. |
| pr_SAK_012 | GTCATCGTCGTACAGATCC | Primer to linearize pTRC to excise spacer coding region. |
| pr_SAK_047 | ATGCAAATGAACCGGCTTACTACGAAGGCGGC | Fwd primer to introduce mismatch of segment 2 on pTarget-cn20 |
| pr_SAK_048 | ATGTTACATCGATAAGCTTG | Rev primer to introduce mismatch of segment 2 on pTarget-cn20 |
| DNA Templates for in vitro transcription of Nes target RNA used in Figure 9 are reported in the supplement of Nasef et al. RNA 2019. DOI: 10.1261/rna.070417.119 | | |
| pmRNA01-F | CUUUGUACUGAUGAUUUAUAUACUUCGGCAUACGUUCUCUAAA | Nes target sequence, labeled with 5’ fluorescein |
| pmRNA01-FMM1 | CUUUGUACUGAUGAUUUAUAUACUUCGGCUAUGCAUCUCUAAA | Nes target sequence, with nucleotides +1 to +6 converted to their base-pair partner, labeled with a 5’ fluorescein |
| pmRNA01-FMM2 | CUUUGUACUGAUGAUUUAUAUACAAGCCGAUACGUUCUCUAAA | Nes target sequence, with nucleotides +7 to +12 converted to their base-pair partner, labeled with a 5’ fluorescein |
| pmRNA-FNC | GCUGACAUUAAGAUUACUAUUUAUUAUCCUUGCGAUUUCUACG | Non-complementary RNA target sequence, labeled with a 5’ fluorescein |
| Plasmid sequences | | |
| >pTarget-nes (4447 bp)  GTTTGACAGCTTATCATCGACTGCACGGTGCACCAATGCTTCTGGCGTCAGGCAGCCATCGGAAGCTGTGGTATGGCTGTGCAGGTCGTAAATCACTGCATAATTCGTGTCGCTCAAGGCGCACTCCCGTTCTGGATAATGTTTTTTGCGCCGACATCATAACGGTTCTGGCAAATATTCTGAAATGAGCTGTTGACAATTAATCATCCGGCTCGTATAATGTGTGGAATTGTGAGCGGATAACAATTTCACACAGGAAACAGCGCCGCTGAGAAAAAGCGAAGCGGCACTGCTCTTTAACAATTTATCAGACAATCTGTGTGGGCACTCGACCGGAATTATCGATTAACTTTATTATTAAAAATTAAAGAGGTATATATTAATGTATCGATTAAATAAGGAGGAATAAACCATGGGGGGTTCTCATCATCATCATCATCATGGTATGGCTAGCATGACTGGTGGACAGCAAATGGGTCGGGATCTGTACGACGATGACGATAAGGATCCAACCCTTTTCCAAGCTTCTTTGTACTGATGATTTATATACTTCGGCATACGTCAAGAGCAGCATGCTTCCAAGGCGAATTCGAAGCTTGGCTGTTTTGGCGGATGAGAGAAGATTTTCAGCCTGATACAGATTAAATCAGAACGCAGAAGCGGTCTGATAAAACAGAATTTGCCTGGCGGCAGTAGCGCGGTGGTCCCACCTGACCCCATGCCGAACTCAGAAGTGAAACGCCGTAGCGCCGATGGTAGTGTGGGGTCTCCCCATGCGAGAGTAGGGAACTGCCAGGCATCAAATAAAACGAAAGGCTCAGTCGAAAGACTGGGCCTTTCGTTTTATCTGTTGTTTGTCGGTGAACGCTCTCCTGAGTAGGACAAATCCGCCGGGAGCGGATTTGAACGTTGCGAAGCAACGGCCCGGAGGGTGGCGGGCAGGACGCCCGCCATAAACTGCCAGGCATCAAATTAAGCAGAAGGCCATCCTGACGGATGGCCTTTTTGCGTTTCTACAAACTCTTTTGTTTATTTTTCTAAATACATTCAAATATGTATCCGCTCATGAGACAATAACCCTGATAAATGCTTCAATAATATTGAAAAAGGAAGAGTATGAGTATTCAACATTTCCGTGTCGCCCTTATTCCCTTTTTTGCGGCATTTTGCCTTCCTGTTTTTGCTCACCCAGAAACGCTGGTGAAAGTAAAAGATGCTGAAGATCAGTTGGGTGCACGAGTGGGTTACATCGAACTGGATCTCAACAGCGGTAAGATCCTTGAGAGTTTTCGCCCCGAAGAACGTTTTCCAATGATGAGCACTTTTAAAGTTCTGCTATGTGGCGCGGTATTATCCCGTGTTGACGCCGGGCAAGAGCAACTCGGTCGCCGCATACACTATTCTCAGAATGACTTGGTTGAGTACTCACCAGTCACAGAAAAGCATCTTACGGATGGCATGACAGTAAGAGAATTATGCAGTGCTGCCATAACCATGAGTGATAACACTGCGGCCAACTTACTTCTGACAACGATCGGAGGACCGAAGGAGCTAACCGCTTTTTTGCACAACATGGGGGATCATGTAACTCGCCTTGATCGTTGGGAACCGGAGCTGAATGAAGCCATACCAAACGACGAGCGTGACACCACGATGCCTGTAGCAATGGCAACAACGTTGCGCAAACTATTAACTGGCGAACTACTTACTCTAGCTTCCCGGCAACAATTAATAGACTGGATGGAGGCGGATAAAGTTGCAGGACCACTTCTGCGCTCGGCCCTTCCGGCTGGCTGGTTTATTGCTGATAAATCTGGAGCCGGTGAGCGTGGGTCTCGCGGTATCATTGCAGCACTGGGGCCAGATGGTAAGCCCTCCCGTATCGTAGTTATCTACACGACGGGGAGTCAGGCAACTATGGATGAACGAAATAGACAGATCGCTGAGATAGGTGCCTCACTGATTAAGCATTGGTAACTGTCAGACCAAGTTTACTCATATATACTTTAGATTGATTTAAAACTTCATTTTTAATTTAAAAGGATCTAGGTGAAGATCCTTTTTGATAATCTCATGACCAAAATCCCTTAACGTGAGTTTTCGTTCCACTGAGCGTCAGACCCCGTAGAAAAGATCAAAGGATCTTCTTGAGATCCTTTTTTTCTGCGCGTAATCTGCTGCTTGCAAACAAAAAAACCACCGCTACCAGCGGTGGTTTGTTTGCCGGATCAAGAGCTACCAACTCTTTTTCCGAAGGTAACTGGCTTCAGCAGAGCGCAGATACCAAATACTGTCCTTCTAGTGTAGCCGTAGTTAGGCCACCACTTCAAGAACTCTGTAGCACCGCCTACATACCTCGCTCTGCTAATCCTGTTACCAGTGGCTGCTGCCAGTGGCGATAAGTCGTGTCTTACCGGGTTGGACTCAAGACGATAGTTACCGGATAAGGCGCAGCGGTCGGGCTGAACGGGGGGTTCGTGCACACAGCCCAGCTTGGAGCGAACGACCTACACCGAACTGAGATACCTACAGCGTGAGCTATGAGAAAGCGCCACGCTTCCCGAAGGGAGAAAGGCGGACAGGTATCCGGTAAGCGGCAGGGTCGGAACAGGAGAGCGCACGAGGGAGCTTCCAGGGGGAAACGCCTGGTATCTTTATAGTCCTGTCGGGTTTCGCCACCTCTGACTTGAGCGTCGATTTTTGTGATGCTCGTCAGGGGGGCGGAGCCTATGGAAAAACGCCAGCAACGCGGCCTTTTTACGGTTCCTGGCCTTTTGCTGGCCTTTTGCTCACATGTTCTTTCCTGCGTTATCCCCTGATTCTGTGGATAACCGTATTACCGCCTTTGAGTGAGCTGATACCGCTCGCCGCAGCCGAACGACCGAGCGCAGCGAGTCAGTGAGCGAGGAAGCGGAAGAGCGCCTGATGCGGTATTTTCTCCTTACGCATCTGTGCGGTATTTCACACCGCATATGGTGCACTCTCAGTACAATCTGCTCTGATGCCGCATAGTTAAGCCAGTATACACTCCGCTATCGCTACGTGACTGGGTCATGGCTGCGCCCCGACACCCGCCAACACCCGCTGACGCGCCCTGACGGGCTTGTCTGCTCCCGGCATCCGCTTACAGACAAGCTGTGACCGTCTCCGGGAGCTGCATGTGTCAGAGGTTTTCACCGTCATCACCGAAACGCGCGAGGCAGCAGATCAATTCGCGCGCGAAGGCGAAGCGGCATGCATTTACGTTGACACCATCGAATGGTGCAAAACCTTTCGCGGTATGGCATGATAGCGCCCGGAAGAGAGTCAATTCAGGGTGGTGAATGTGAAACCAGTAACGTTATACGATGTCGCAGAGTATGCCGGTGTCTCTTATCAGACCGTTTCCCGCGTGGTGAACCAGGCCAGCCACGTTTCTGCGAAAACGCGGGAAAAAGTGGAAGCGGCGATGGCGGAGCTGAATTACATTCCCAACCGCGTGGCACAACAACTGGCGGGCAAACAGTCGTTGCTGATTGGCGTTGCCACCTCCAGTCTGGCCCTGCACGCGCCGTCGCAAATTGTCGCGGCGATTAAATCTCGCGCCGATCAACTGGGTGCCAGCGTGGTGGTGTCGATGGTAGAACGAAGCGGCGTCGAAGCCTGTAAAGCGGCGGTGCACAATCTTCTCGCGCAACGCGTCAGTGGGCTGATCATTAACTATCCGCTGGATGACCAGGATGCCATTGCTGTGGAAGCTGCCTGCACTAATGTTCCGGCGTTATTTCTTGATGTCTCTGACCAGACACCCATCAACAGTATTATTTTCTCCCATGAAGACGGTACGCGACTGGGCGTGGAGCATCTGGTCGCATTGGGTCACCAGCAAATCGCGCTGTTAGCGGGCCCATTAAGTTCTGTCTCGGCGCGTCTGCGTCTGGCTGGCTGGCATAAATATCTCACTCGCAATCAAATTCAGCCGATAGCGGAACGGGAAGGCGACTGGAGTGCCATGTCCGGTTTTCAACAAACCATGCAAATGCTGAATGAGGGCATCGTTCCCACTGCGATGCTGGTTGCCAACGATCAGATGGCGCTGGGCGCAATGCGCGCCATTACCGAGTCCGGGCTGCGCGTTGGTGCGGATATCTCGGTAGTGGGATACGACGATACCGAAGACAGCTCATGTTATATCCCGCCGTTAACCACCATCAAACAGGATTTTCGCCTGCTGGGGCAAACCAGCGTGGACCGCTTGCTGCAACTCTCTCAGGGCCAGGCGGTGAAGGGCAATCAGCTGTTGCCCGTCTCACTGGTGAAAAGAAAAACCACCCTGGCGCCCAATACGCAAACCGCCTCTCCCCGCGCGTTGGCCGATTCATTAATGCAGCTGGCACGACAGGTTTCCCGACTGGAAAGCGGGCAGTGAGCGCAACGCAATTAATGTAAGTTAGCGCGAATTGATCTG | | |
| >pACYC-CRISPR-spc1 (10792 bp)  ATGCACGAACCCCCCGTTCAGTCCGACCGCTGCGCCTTATCCGGTAACTATCGTCTTGAGTCCAACCCGGAAAGACATGCAAAAGCACCACTGGCAGCAGCCACTGGTAATTGATTTAGAGGAGTTAGTCTTGAAGTCATGCGCCGGTTAAGGCTAAACTGAAAGGACAAGTTTTGGTGACTGCGCTCCTCCAAGCCAGTTACCTCGGTTCAAAGAGTTGGTAGCTCAGAGAACCTTCGAAAAACCGCCCTGCAAGGCGGTTTTTTCGTTTTCAGAGCAAGAGATTACGCGCAGACCAAAACGATCTCAAGAAGATCATCTTATTAATCAGATAAAATATTTCTAGATTTCAGTGCAATTTATCTCTTCAAATGTAGCACCTGAAGTCAGCCCCATACGATATAAGTTGTAATTCTCATGTTTGACAGCCTTCAGATCCGATAGACTAGCCGCTGGTAATAATACGACTCACTATAGGGAGAGAATTCTAAGACCGAAAGTCGGAAACAAAGAGGATTTATATGATCAACAAAATCACCGTGGAACTGGATCTGCCGGAAAGCATTCGTTTTCAGTATCTGGGTAGCGTTCTGCATGGTGTTCTGATGGATTATCTGAGTGATGATATTGCAGATCAGCTGCATCACGAATTTGCATATAGTCCGCTGAAACAGCGCATCTACCACAAAAACAAAAAAATCATCTGGGAAATCGTGTGCATGAGCGATAACCTGTTTAAAGAAGTGGTGAAACTGTTTAGCAGCAAAAATAGCCTGCTGCTGAAATATTACCAGACCAACATTGATATCCAGAGCTTCCAGATCGAAAAAATCAATGTGCAGAACATGATGAATCAGCTGCTGCAGGTTGAGGATCTGAGCCGTTATGTTCGTCTGAACATTCAGACCCCGATGAGCTTCAAATATCAGAACAGCTATATGATCTTCCCGGATGTGAAACGTTTTTTCCGCAGCATTATGATTCAGTTCGATGCCTTTTTTGAAGAATACCGCATGTACGATAAAGAAACCCTGAACTTCCTGGAAAAAAACGTGAACATCGTGGATTATAAACTGAAAAGCACCCGCTTTAATCTGGAAAAAGTTAAAATTCCGAGCTTTACCGGTGAGATCGTGTTCAAAATCAAAGGTCCGCTGCCGTTTCTGCAGCTGACCCATTTTCTGCTGAAATTTGGTGAATTTAGCGGCAGCGGTATTAAAACCAGCCTGGGTATGGGTAAATATAGCATCATCTAAAAGCTTTCCTGTGAGCAGCGAAAGCCTAGCATAACCCCTTGGGGCCTCTAAACGGGTCTTGAGGGGTTTTTTGTTATACGCGAGATAATCACTTGCATAGCTGCGTATGGAGGAAGCAACTCTTGAGTGTTAATATGTTGACCCCTGTATTAGGGATGCGGGTAGTAGATGTGGGCAGAGACACCCACACTGCCAGATCTTAATACGACTCACTATAGGGAGACCATGGGATCGATACCCACCCCGAAGAAAAGGGGACGAGAACACGTATGCCGAAGTATATAAATCATCAGTACAAAGGATCGATACCCACCCCGAAGAAAAGGGGACGAGAACCTCGAGGCTGTGGTCTAGACATTCCATACATATCGGGGGGGTAGGGGTTTTTTGTGTGCCTCTAGTGGCTGGCTAAGAATAATACGACTCACTATAGGGAGAGGATCCATAAAGGAGGTAAATAATGAACAAAAAAAACATCCTGATGTATGGCAGCCTGCTGCATGATATTGGCAAAATTATCTATCGTAGCGGTGATCATACCTTTAGCCGTGGCACCCATAGCAAACTGGGTCATCAGTTTCTGAGCCAGTTTAGCGAATTTAAAGATAACGAAGTGCTGGATAACGTGGCCTATCATCATTATAAAGAACTGGCAAAAGCCAACCTGGATAATGATAATACCGCCTACATTACCTATATCGCCGATAATATTGCAAGCGGTATTGATCGTCGCGATATTATTGAAGAGGGTGATGAAGAATATGAGAAACAACTGTTCAACTTCGATAAATACACACCGCTGTATAGCGTGTTTAACATTGTGAATAGCGAAAAACTGAAACAGACCAACGGCAAATTCAAATTTAGCAACGAAAGCAACATCGAATACCCGAAAACCGAAAACATTCAGTATAGCAGCGGTAATTATACCACCCTGATGAAAGATATGAGCCATGATCTGGAACATAAACTGAGCATTAAAGAAGGCACCTTTCCGAGTCTGCTGCAGTGGACCGAAAGCCTGTGGCAGTATGTTCCGAGCAGCACCAATAAAAACCAGCTGATTGATATCAGCCTGTATGACCATAGCCGTATTACCTGTGCAATTGCCAGCTGCATTTTTGATTATCTGAACGAGAACAACATCCACAACTATAAAGATGAACTGTTTAGCAAATATGAAAACACCAAATCCTTTTATCAGAAAGAGGCATTTCTGCTGCTGAGCATGGATATGAGCGGTATTCAGGATTTCATCTATAACATTAGCGGTAGCAAAGCACTGAAAAGCCTGCGTAGCCGTAGCTTTTATCTGGAACTGATGCTGGAAGTTATTGTTGATCAGCTGCTGGAACGCCTGGAACTGGCACGTGCAAATCTGCTGTATACCGGTGGTGGTCATGCATATCTGCTGGTTAGCAATACCGACAAAGTGAAAAAAAAAATCACCCAGTTCAACAACGAACTGAAAAAATGGTTTATGAGCGAGTTTACCACCGATCTGAGCCTGTCAATGGCATTTGAAAAATGTAGTGGTGATGACCTGATGAATACCAGCGGCAATTATCGTACCATTTGGCGTAATGTTAGCAGCAAACTGAGCGATATTAAAGCCCACAAATATAGCGCAGAGGACATTCTGAAACTGAACCATTTTCATAGTTATGGCGATCGCGAATGTAAAGAATGTCTGCGTAGCGATATTGACATTAACGATGATGGTCTGTGTAGCATTTGCGAAGGCATTATTAACATCAGCAATGATCTGCGCGACAAATCGTTTTTTGTGCTGAGCGAAACCGGTAAACTGAAAATGCCGTTTAACAAATTCATCAGCGTGATCGATTATGAAGAGGCCGAAATGCTGGTTCAGAATAATAACCAGGTTCGCATCTATAGCAAAAACAAACCGTATATTGGCATTGGCATTAGCACCAATCTGTGGATGTGTGATTATGATTATGCAAGCCAGAATCAGGATATGCGCGAAAAAGGTATTGGTAGCTATGTTGATCGTGAAGAAGGTGTTAAACGTCTGGGTGTTGTTCGTGCAGATATTGATAATCTGGGTGCAACCTTTATTAGCGGCATTCCGGAAAAATACAATAGCATTAGCCGTACCGCAACCCTGAGCCGTCAGCTGAGTCTGTTCTTTAAATACGAGCTGAACCATCTGCTGGAAAACTATCAGATTACCGCAATTTATAGTGGCGGAGATGACCTGTTTCTGATTGGTGCATGGGATGATATTATCGAAGCGAGCATTTACATCAACGATAAATTCAAAGAGTTTACCCTGGACAAACTGACCCTGAGTGCCGGTGTTGGCATGTTTAGCGGTAAATATCCGGTTAGCAAAATGGCCTTTGAGACAGGTCGTCTGGAAGAGGCAGCAAAAACTGGCGAAAAAAACCAGATTAGTCTGTGGCTGCAAGAGAAAGTGTATAACTGGGATGAGTTCAAAAAAAACATTCTGGAAGAGAAACTGCTGGTTCTGCAGCAGGGTTTTAGCCAGACCGATGAACATGGTAAAGCCTTCATTTACAAAATGCTGGCACTGCTGCGTAATAACGAAGCAATTAACATTGCACGTCTGGCATACCTGCTGGCACGTAGTAAAATGAATGAAGATTTCACCAGCAAAATCTTTAACTGGGCACAGAACGACAAAGACAAAAATCAACTGATTACAGCCCTGGAATACTATATCTATCAGATCCGTGAAGCCGACTAACATATGGCTGCGTGGTCAAATGTGCGTACCCTAACCCCTTCCCCGGTCAATCGGGGCGGATGGGGTTTTTTGTGCGTACTTCATTATGTATATTAATACGACTCACTATAGGGAGAAGATCTATAAAGGAGGTAAATAATGGGTCACCACCATCATCACCATAGCGGTGGAATTCTGGCCAAAACCAAAAGCGGCAAAACCATTGATCTGACCTTTGCACATGAAGTGGTTAAAAGCAATGTGAAAAACGTGAAAGACCGCAAAGGCAAAGAAAAACAGGTTCTGTTTAATGGTCTGACCACCAGTAAACTGCGTAATCTGATGGAACAGGTTAATCGCCTGTATACCATTGCCTTTAATAGCAATGAAGATCAGCTGAACGAAGAGTTTATCGATGAACTGGAATATCTGAAAATCAAATTCTACTATGAAGCCGGTCGTGAGAAAAGCGTTGATGAGTTTCTGAAAAAAACCCTGATGTTCCCGATTATTGATCGCGTGATCAAAAAAGAAAGCAAAAAATTCTTCCTGGACTACTGCAAATATTTCGAAGCACTGGTTGCATACGCCAAATATTACCAGAAAGAGGACTAAACGCGTGCTGCGTGGTCAAATGTGCGTAGACCAACCCCTTGCGGCCTCAATCGGGGGGGATGGGGTTTTTTGTCAGGCAAGTCTCAGCTGGTTTAATACGACTCACTATAGGGAGAGAATTCATAAAGGAGGTAAATAATGGGGTTTTTTGTCAGGCAAGTCTCAGCTGGTTTAATACGACTCACTATAGGGAGAGAATTCCCCAGCAGTATAACAGGAGGACACCAGATGTACAGCAAAATCAAAATCAGCGGCACCATTGAAGTTGTTACCGGTCTGCATATTGGTGGTGGTGGCGAAAGCAGCATGATTGGTGCAATTGATAGTCCGGTTGTTCGTGATCTGCAGACCAAACTGCCGATTATTCCGGGTAGCAGCATTAAAGGTAAAATGCGTAATCTGCTGGCCAAACACTTTGGCCTGAAAATGAAACAAGAAAGCCATAACCAGGATGATGAACGTGTTCTGCGTCTGTTTGGTAGCAGCGAAAAAGGTAATATTCAGCGTGCTCGCCTGCAGATTAGTGATGCATTTTTTAGCGAAAAAACCAAAGAACACTTCGCCCAGAATGATATTGCATACACCGAAACCAAATTCGAGAATACCATTAATCGTCTGACCGCAGTTGCAAATCCGCGTCAGATTGAACGTGTGACCCGTGGTAGCGAATTTGACTTTGTGTTTATCTATAACGTGGATGAAGAGTCCCAGGTGGAAGATGATTTTGAAAACATTGAGAAAGCGATCCATCTGCTGGAAAATGATTATCTGGGTGGCGGTGGTACACGTGGTAATGGTCGTATTCAGTTTAAAGACACCAACATTGAAACCGTGGTGGGTGAATATGATAGCACCAATCTGAAAATCAAATAAAAGCTTACCTGGAGATCAAGGAGATTACTCTAACCCCATCGGCCGTCTTAGGGGTTTTTTGTCCTGTGTTAGCTGGAGGGTATAATACGACTCACTATAGGGAGACCCGGGATAAAGGAGGTAAATAATGACCCTGGCAACCAAAGTTTTTAAACTGAGCTTTAAAACACCGGTGCATTTCGGTAAAAAACGTCTGAGTGATGGTGAAATGACCATTACCGCAGATACCCTGTTTAGCGCACTGTTTATTGAAACCCTGCAGCTGGGTAAAGATACCGATTGGCTGCTGAATGATCTGATTATTAGCGATACCTTTCCGTATGAGAACGAGCTGTATTATCTGCCGAAACCGCTGATTAAAATCGACAGCAAAGAAGAGGATAACCACAAAGCCTTCAAAAAACTGAAATATGTGCCGGTGCATCACTATAACCAGTATCTGAATGGTGAACTGAGCGCAGAAGATGCAACCGATCTGAATGATATTTTCAACATCGGCTATTTCAGCCTGCAGACCAAAGTTAGCCTGATTGCACAAGAAACCGATAGCAGCGCAGATAGCGAACCGTATAGCGTTGGCACCTTTACCTTTGAACCGGAAGCAGGTCTGTATTTTATCGCAAAAGGTAGCGAAGAAACCCTGGATCATCTGAATAACATTATGACCGCACTGCAGTATAGCGGTCTGGGTGGTAAACGTAATGCAGGTTATGGTCAGTTTGAGTACGAAATCATTAATAACCAGCAGCTGAGCAAACTGCTGAATCAGAATGGTAAACATAGCATTCTGCTGAGCACCGCAATGGCAAAAAAAGAAGAAATTGAAAGCGCACTGAAAGAGGCACGTTATATTCTGACCAAACGTAGCGGTTTTGTTCAGAGCACCAATTATAGCGAAATGCTGGTGAAAAAAAGCGACTTCTATAGCTTTAGCAGCGGCAGCGTTTTCAAAAACATTTTTAACGGCGATATCTTCAACGTGGGCCATAATGGCAAACATCCGGTTTATCGTTATGCTAAACCGCTGTGGCTGGAAGTTTAATCATGATTTCTTGTCGAACTGGACAGTAGCAGAACCGCTAACGGGGGCGAAGGGGTTTTTTGTGACATACGAGCTGATTGAACTAATACGACTCACTATAGGGAGAGGTACCATAAAGGAGGTAAATAATGACCATCAAAAACTATGAGGTGGTGATTAAAACCCTGGGTCCGATTCATATTGGTAGCGGTCAGGTTATGAAAAAACAGGATTATATCTACGACTTTTATAACAGCAAAGTGTATATGATCAACGGCAACAAACTGGTGAAATTTCTGAAACGCAAAAACCTGCTGTATACCTATCAGAACTTTCTGCGTTATCCGCCTAAAAATCCGCGTGAAAATGGTCTGAAAGATTATCTGGATGCCCAGAATGTTAAACAGAGCGAATGGGAAGCATTTGTGAGCTATAGCGAAAAAGTGAACCAGGGCAAAAAATACGGTAATACCCGTCCGAAACCGCTGAATGATCTGCATCTGATGGTTCGTGATGGTCAGAATAAAGTTTATCTGCCTGGTAGCAGCATTAAAGGTGCAATTAAAACCACCCTGGTGAGCAAATATAACAACGAAAAAAACAAAGATATCTATAGCAAAATCAAAGTGAGCGATAGCAAACCGATTGATGAAAGCAATCTGGCCATCTATCAGAAAATCGACATCAACAAAAGCGAGAAAAGCATGCCGCTGTATCGTGAATGTATTGATGTGAACACCGAGATCAAATTCAAACTGACCATCGAGGATGAAATCTACAGCATCAATGAAATCGAACAGAGCATCCAGGACTTCTATAAAAACTACTATGATAAATGGCTGGTCGGCTTTAAAGAAACCAAAGGTGGTCGTCGTTTTGCACTGGAAGGTGGTATTCCGGATGTTCTGAATCAAAACATTCTGTTTCTGGGTGCAGGCACCGGTTTTGTGAGCAAAACCACACATTATCAGCTGAAAAATCGCAAACAGGCCAAACAGGATAGCTTTGAAATTCTGACGAAAAAATTCCGTGGCACCTACGGCAAAATGAAAGAAATTCCGAGCAATGTTCCGGTTGCACTGAAAGGCACCACCAATCAGAGCCGTCATACCAGCTATCAGCAGGGTATGTGTAAAGTTAGCTTTCAAGAACTGAACAACGAGGTGCTGTAACCTAGGCGCTTCAACGGAACGGATCTTACATATCGGGGGGGTAGGGGTTTTTTGTCTCGGAGACCAAGTAGGGCATAATACGACTCACTATAGGGAGACTATGGATAAAGGAGGTAAATAATGAAAATCCTGTTTAGCCCGATTGGTAATAGCGATCCGTGGCGTAATGATCGTGATGGTGCAATGCTGCATATTGTGCGTCATTATAATCTGGATAAAGTGGTGCTGTATTTCACCCGTACCATTTGGGAAGGTAATGAAAATCGCAAAGGCCACAAAATCTATGAATGGGAGAAAATTATCCAGACCGTTAGCCCGAATACCGAAGTGGAAATTATCATTGAAAATGTGGATAACGCCCAGGATTACGATGTGTTCAAAGAGAAATTCCATAAATATCTGAAAATCATCGAAGATAGCTACGAGGATTGCGAAATTATTCTGAATGTTACCAGCGGTACACCGCAGATGGAAAGCACCCTGTGTCTGGAATATATTGTTTACCCGGAAAACAAAAAATGCGTTCAGGTTAGCACCCCGACCAAAGATAGCAATGCAGGTATTGAATATAGCAACCCGAAAGACAAAGTGGAAGAATTTGAAATCGTGAACGAAGTCGAGAAAAAAAGCGAAAAACGCTGCAAAGAAATCAACATTCTGAGCTTTCGTGAAGCCATGATTCGTAGCCAGATTCTGGGTCTGATTGATAACTATGATTATGAAGGTGCCCTGAATCTGGTGAGCAATCAGAAAAGTTTTCGCAATGGTAAACTGCTGCGTAAAAAACTGCTGAGCCTGACCAAACAAATCAAAACCCATGAAGTTTTCCCGGAAATCAACGAAAAATATCGTGATGACGCCCTGAAAAAATCCCTGTTTCATTATCTGCTGCTGAACATGCGTTATAATCGTCTGGATGTTGCAGAAACCCTGATTCGTGTTAAAAGCATTGCAGAGTTTATCCTGAAAACCTACATCGAAATTCATTGGCCGACCCTGATTATTGAGAAAGATGGTAAACCGTATCTGAACGATGAAGATAATCTGTCCTTCGTGTACAAATACAACCTGCTGCTGGAAAAACGCAAACAGAATTTTGATGTTAGCCGTATTCTGGGCCTGCCTGCATTTATTGATATTCTGACCATTCTGGAACCGAATAGCCAGCTGCTGAAAGAAGTTAACGCAGTTAACGATATTAATGGCCTGCGTAATAGCATTGCCCATAACCTGGATACCCTGAACCTGGACAAAAATAAAAACTACAAAAAAATCATGCTGAGCGTGGAAGCCATCAAAAATATGCTGCACATTAGCTTCCCTGAAATCGAGGAAGAGGATTATAACTATTTTGAAGAGAAAAACAAAGAATTTAAAGAACTGCTGTAACTCGAGAGGTTACAGCCTGCATAATGTAGCATAACCCCTTGGGGCCTCTAAACGGGTCTTGAGGGGTTTTTTGTGCCTATAGTTTGAAGCAGAATCGAATTTCTGCCATTCATCCGCTTATTATCACTTATTCAGGCGTAGCACCAGGCGTTTAAGGGCACCAATAACTGCCTTACAAAAAACCCCTAGCCGCCCGATAAGAGCGGGCTAGGGGTTCGAGTAAAAAAAATTACGCCCCGCCCTGCCACTCATCGCAGTACTGTTGTAATTCATTAAGCATTCTGCCGACATGGAAGCCATCACAGACGGCATGATGAACCTGAATCGCCAGCGGCATCAGCACCTTGTCGCCTTGCGTATAATATTTGCCCATCGTGAAAACGGGGGCGAAGAAGTTGTCCATATTGGCCACGTTTAAATCAAAACTGGTGAAACTCACCCAGGGATTGGCTGAAACGAAAAACATATTCTCAATAAACCCTTTAGGGAAATAGGCCAGGTTTTCACCGTAACACGCCACATCTTGCGAATATATGTGTAGAAACTGCCGGAAATCGTCGTGGTATTCACTCCAGAGCGATGAAAACGTTTCAGTTTGCTCATGGAAAACGGTGTAACAAGGGTGAACACTATCCCATATCACCAGCTCACCGTCTTTCATTGCCATACGGAACTCCGGGTGAGCATTCATCAGGCGGGCAAGAATGTGAATAAAGGCCGGATAAAACTTGTGCTTATTTTTCTTTACGGTCTTTAAAAAGGCCGTAATATCCAGCTGAACGGTCTGGTTATAGGTACATTGAGCAACTGACTGAAATGCCTCAAAATGTTCTTTACGATGCCATTGGGATATATCAACGGTGGTATATCCAGTGATTTTTTTCTCCATTTTAGCTTCCTTAGCTCCTGAAAATCTCGATAACTCAAAAAATACGCCCGGTAGTGATCTTATTTCATTATGGTGAAAGTTGGAACCTCTTACGTGCCGATCAAGGTCTCATTTTCGCCAAAAGTTGGCCCAGGGCTTCCCGGTATCAACAGGGACACCAGGATTTATTTATTCTGCGAAGTGATCTTCCGTCACAGGTATTTATTCGGCGCAAAGTGCGTCGGGTGATGCTGCCAACTTACTGATTTAGTGTATGATGGTGTTTTTGAGGTGCTCCAGTGGCTTCTGTTTCTATCAGCTGTCCCTCCTGTTCAGCTACTGACGGGGTGGTGCGTAACGGCAAAAGCACCGCCGGACATCAGCGCTAGCGGAGTGTATACTGGCTTACTATGTTGGCACTGATGAGGGTGTCAGTGAAGTGCTTCATGTGGCAGGAGAAAAAAGGCTGCACCGGTGCGTCAGCAGAATATGTGATACAGGATATATTCCGCTTCCTCGCTCACTGACTCGCTACGCTCGGTCGTTCGACTGCGGCGAGCGGAAATGGCTTACGAACGGGGCGGAGATTTCCTGGAAGATGCCAGGAAGATACTTAACAGGGAAGTGAGAGGGCCGCGGCAAAGCCGTTTTTCCATAGGCTCCGCCCCCCTGACAAGCATCACGAAATCTGACGCTCAAATCAGTGGTGGCGAAACCCGACAGGACTATAAAGATACCAGGCGTTTCCCCCTGGCGGCTCCCTCGTGCGCTCTCCTGTTCCTGCCTTTCGGTTTACCGGTGTCATTCCGCTGTTATGGCCGCGTTTGTCTCATTCCACGCCTGACACTCAGTTCCGGGTAGGCAGTTCGCTCCAAGCTGGACTGT | | |

**
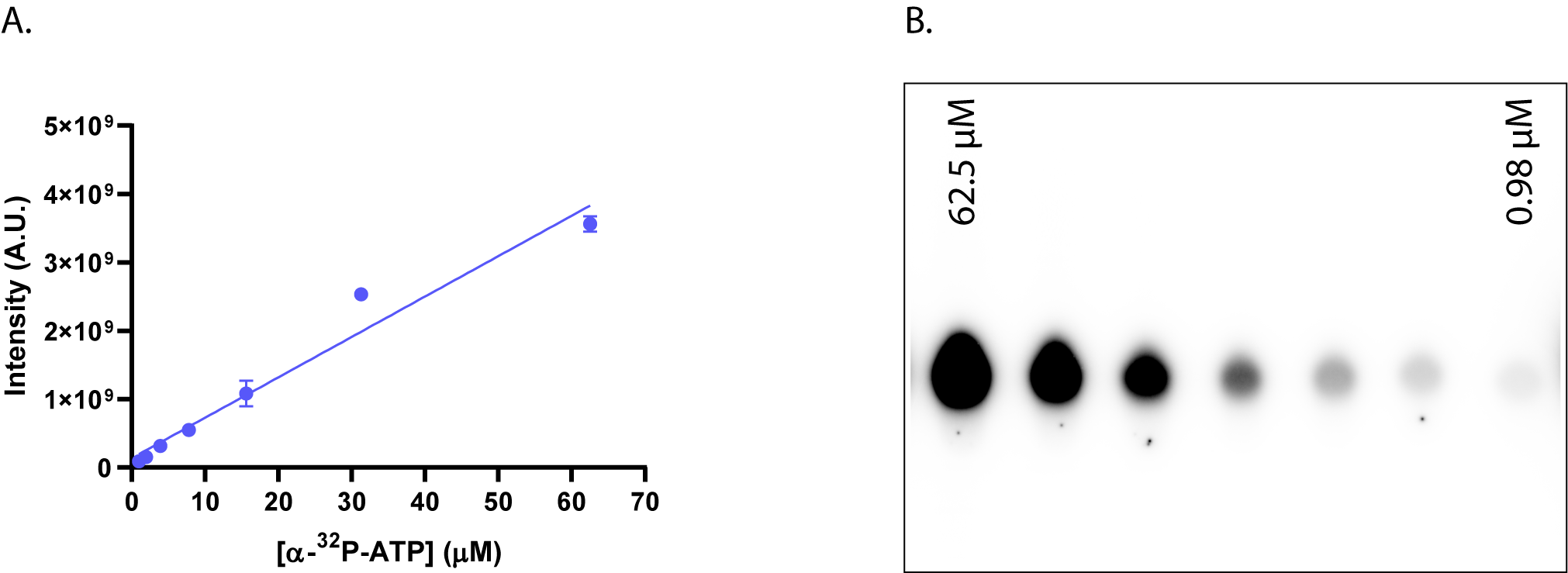
**

**Figure S4. A standard curve of α-^32^P-ATP.** (A) Linear regression of the integrated intensities of α-^32^P-ATP. (B) A representative TLC plate with serial dilutions of α-^32^P-ATP

**
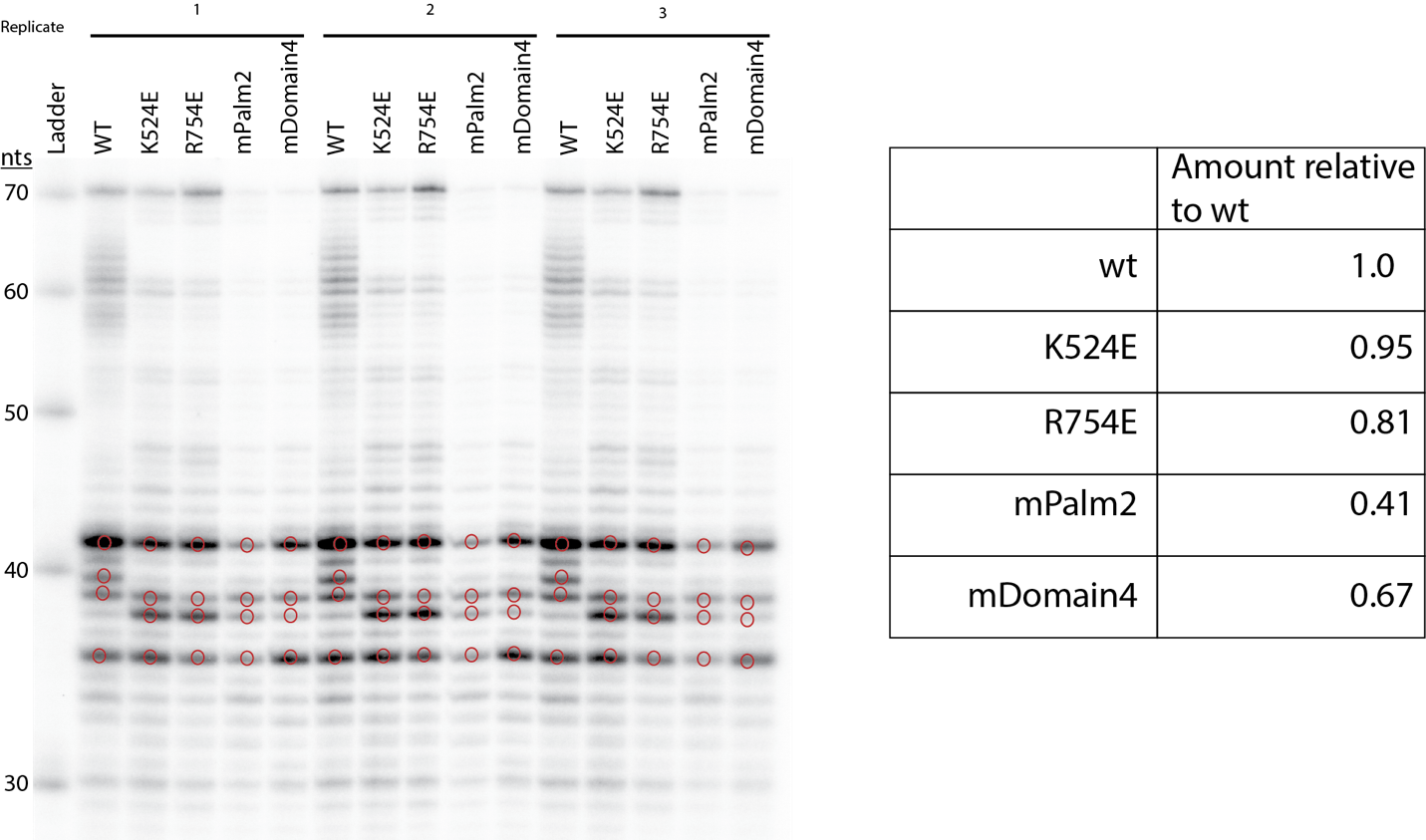
**

**Figure S5. Quantitation of crRNA associated with Cas10-Csm variants.** PAGE separation of crRNA associated with purified wt or mutant Cas10-Csm complexes. Red dots in the image indicate bands that were integrated by ImageQuant software. The mean amount of crRNA from three technical replicates is given in the table expressed in units relative to wt.

**
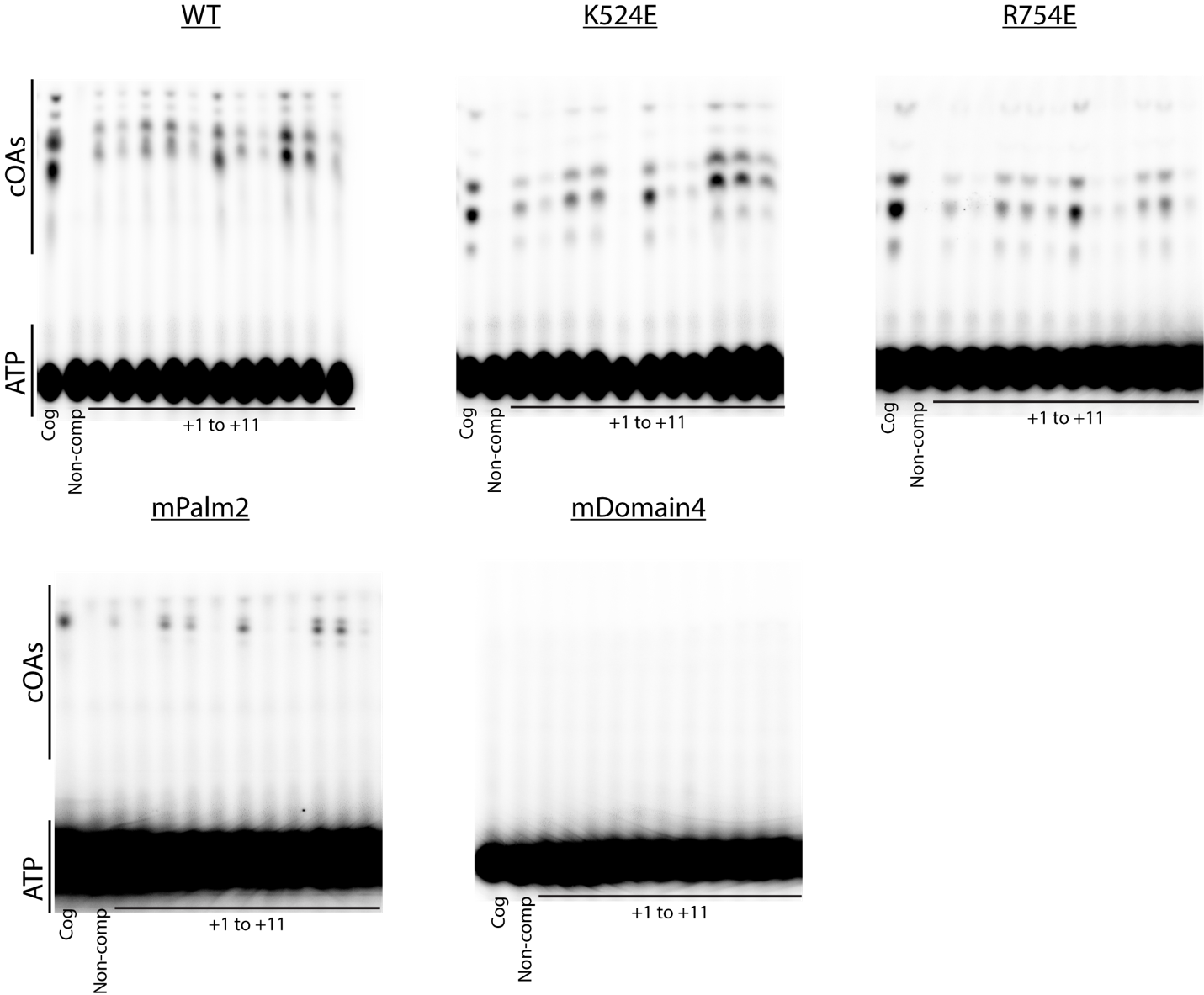
**

**Figure S6. Uncropped images of TLC plates reported in Figure 9.
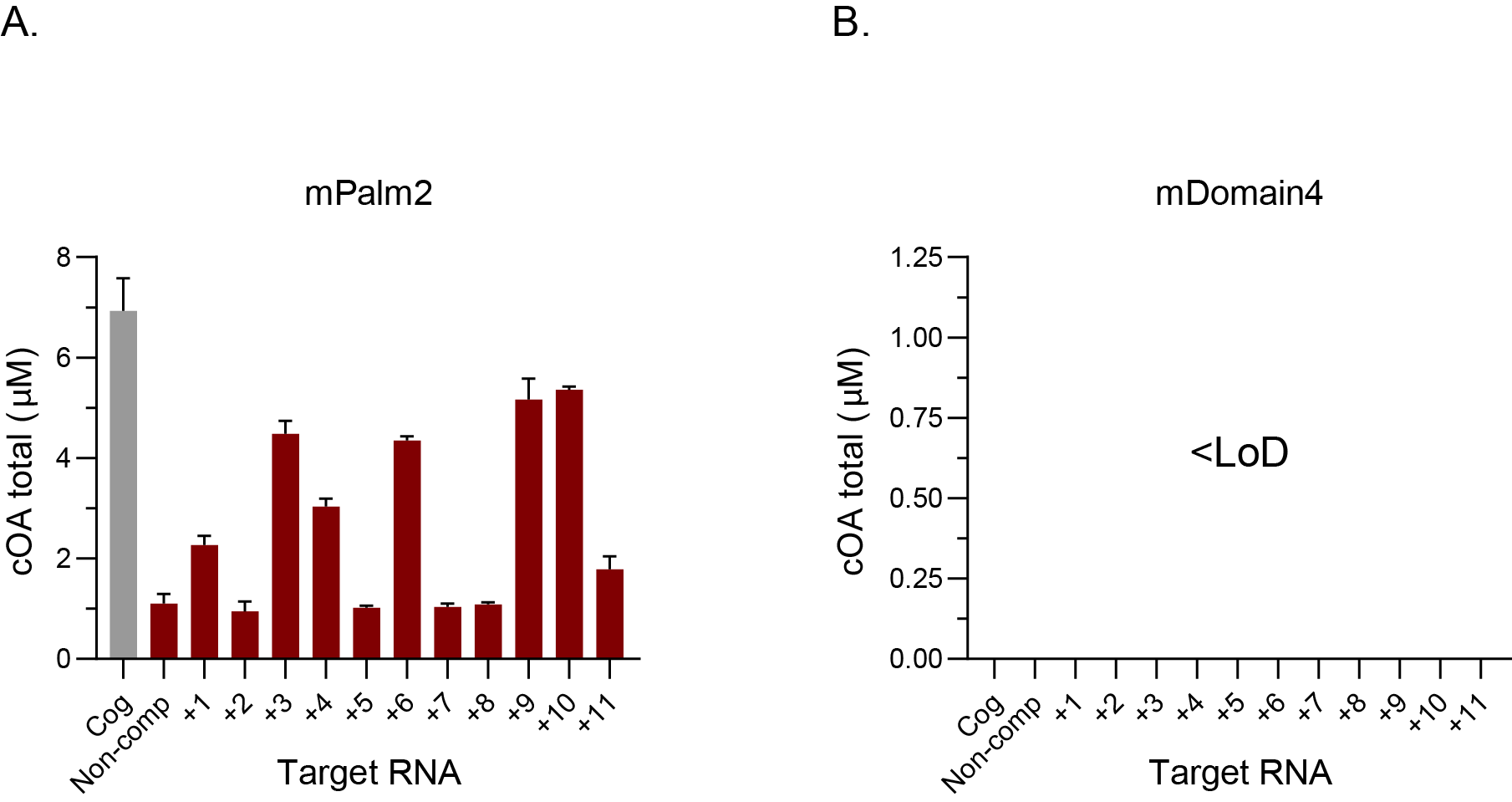
**

**Figure S7. COA synthesis by mPalm2 and mDomain4 Cas10-Csm variants.**
